# MoBioS: Modular Platform Technology for High-Throughput Construction and Characterization of Tunable Transcriptional Biological Sensors

**DOI:** 10.3390/bios13060590

**Published:** 2023-05-30

**Authors:** Wouter Demeester, Jasmine De Baets, Dries Duchi, Marjan De Mey, Brecht De Paepe

**Affiliations:** Centre for Synthetic Biology (CSB), Ghent University, 9000 Ghent, Belgiummarjan.demey@ugent.be (M.D.M.)

**Keywords:** transcriptional biosensors, high-throughput construction, plasmid construction platform, prokaryotes

## Abstract

All living organisms have evolved and fine-tuned specialized mechanisms to precisely monitor a vast array of different types of molecules. These natural mechanisms can be sourced by researchers to build Biological Sensors (BioS) by combining them with an easily measurable output, such as fluorescence. Because they are genetically encoded, BioS are cheap, fast, sustainable, portable, self-generating and highly sensitive and specific. Therefore, BioS hold the potential to become key enabling tools that stimulate innovation and scientific exploration in various disciplines. However, the main bottleneck in unlocking the full potential of BioS is the fact that there is no standardized, efficient and tunable platform available for the high-throughput construction and characterization of biosensors. Therefore, a modular, Golden Gate-based construction platform, called MoBioS, is introduced in this article. It allows for the fast and easy creation of transcription factor-based biosensor plasmids. As a proof of concept, its potential is demonstrated by creating eight different, functional and standardized biosensors that detect eight diverse molecules of industrial interest. In addition, the platform contains novel built-in features to facilitate fast and efficient biosensor engineering and response curve tuning.

## 1. Introduction

The finite resources of our planet have driven us to shift from a petrochemical-based towards a bio-based industry. Industrial biotechnology plays a key role in this transition by providing ways to sustainably produce a broad range of enzymes, pharmaceuticals, foods, fuels and chemical building blocks with numerous applications [1]. The range of possibilities is rapidly expanding, stimulated by the development of modified microorganisms, i.e., Microbial Cell Factories (MCFs), which both efficiently and sustainably produce these molecules of great interest.

The development of these MCFs usually involves multiple iterations of the Design-Build-Test-Learn (DBTL) cycle to find an optimal producer [2]. The current DNA read-and-write technologies allow simultaneous generation of a vast library of MCFs, thereby speeding up the Design and Build phases [3]. However, the Test phase is still limited by laborious, time-consuming and expensive analytical methods, such as high-performance liquid chromatography with UV-detectors (HPLC-UV) and gas chromatography coupled to mass spectrometry (GC-MS), thereby abolishing the high-throughput potential of these libraries of MCF variants. Transcriptional biological sensors, as in vivo, real-time, specific and quantitative detection tools, can rapidly speed up this process by measuring the concentration of a molecule of interest. In combination with high-throughput methods, such as fluorescence-activated cell sorting (FACS), they enable the screening of thousands of variants simultaneously [4,5].

**Figure 1:**
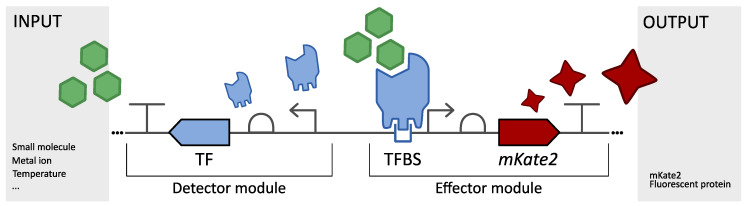
A schematic representation of a transcriptional biological sensor is shown. The detector of this biosensor, a transcription factor (TF), will recognize a certain input, the molecule of interest. This binding results in conformational changes to the TF, leading to differences in affinity for its transcription factor binding site (TFBS), which is located in a promoter region. Changes in this affinity impact promoter activity, which regulates expression of the output. A fluorescent protein, e.g., mKate2, is chosen here for ease of quantification.

A transcriptional biological sensor is a detection tool based on prokaryotic transcription factors (TFs). These proteins are the key component in transcriptional regulatory mechanisms. They enable cells to detect and respond to a whole range of internal and external stimuli, such as antibiotics, fatty acids, secondary metabolites, different carbon sources, CO_2_/O_2_, metal ions, pH, light and temperature [4,6,7,8,9]. Using these TFs, biosensors transform an input, i.e., the presence or absence of a molecule of interest, into an easily detectable output, usually a fluorescent protein, in a concentration-dependent manner [4]. From a bio-engineering point of view, a transcriptional biosensor can be divided into a detector module and an effector module (Figure 1). In the detector module, the transcription factor will specifically bind to the molecule of interest, leading to a conformational change in its protein structure. This structural change impacts the affinity of the protein for its respective transcription factor binding site(s) (TFBS(s)). In the effector module, the interaction of the TF with the TFBS, a 5-to-30 base pair DNA consensus sequence situated in the vicinity of the core promoter, will up- and/or downregulate the corresponding responsive promoter’s activity [8,10]. By using this promoter to control the expression of an output of choice, such as a fluorescent protein, its activity can be externally measured and linked to the input concentration. In this way, MCF variants with diverse productivities can be screened in a high-throughput manner using fluorescent biosensor output [11,12]. In addition, sensing molecules across the pathway of interest can be the starting point for dynamic pathway regulation or biosensor-driven evolution [13,14,15,16,17,18,19]. For these biosensor applications, the biosensor output would be a pathway enzyme or selection marker, respectively, instead of a fluorescent protein. Finally, these biosensors are portable, self-generating, cheap and online, thereby expanding the range of possible applications beyond biotechnology and towards environmental and medical sensing. They are widely used as important and efficient tools for the detection of pathogens, metals and pollutants [6,20,21].

With this plethora of possibilities and applications, it is not surprising that biosensing is seen as one of the ten key technological advances for the next generation of synthetic biology [22]. Despite several biosensors being developed in recent decades, a standardized, modular architecture for constructing these tools is still lacking, thereby limiting expansion of the biosensor portfolio. This hampers the efficient development and portability of biosensors both within and across research groups. Creating a modular architecture with standardized protocols would alleviate these problems by allowing the rapid construction and optimization of multiple biosensors simultaneously. Additionally, standardization of the design and characterization protocols is required to build a functional database with replicable outcomes [23,24].

**Figure 2:**
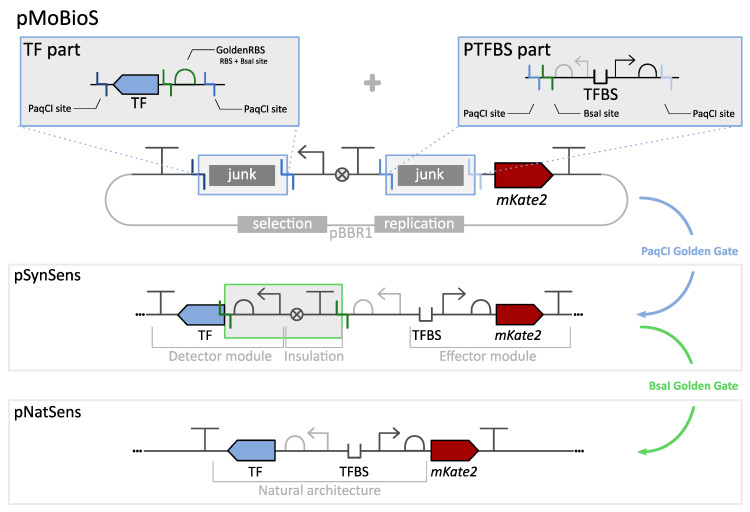
An overview of the MoBioS platform workflow is depicted. Two parts, a transcription factor part (TF, top left) and a promoter with transcription factor binding site part (PTFBS, top right), are flanked by PaqCI Golden Gate sites and inserted into the MoBioS platform in a single Golden Gate reaction. This generates a pSynSens plasmid that consists of an independent detector and effector module. In addition, and if applicable, the platform allows the construction of a biosensor variant that mimics the natural architecture by performing a subsequent BsaI Golden Gate reaction. The GoldenRBS assures that the natural RBS incorporated in the PTFBS is now directly upstream of the start codon of the transcription factor, thereby generating the pNatSens biosensor plasmid.

In this research paper, a modular plug-and-play platform, called MoBioS or Modular Biological Sensors, was developed to speed up the standardized construction of biosensors (Figure 2). Based on a one-pot, one-step Golden Gate reaction, this plasmid-based platform allows the combination of any TF with any TFBS to efficiently screen for the best performing biosensor for any molecule of interest. As a proof of concept, a library of biosensors was created and characterized in *Escherichia coli* based on transcription factors from four different transcription factor families to illustrate the versatility of the developed technology. The result is a new, standardized portfolio of fully characterized biosensors.

## 2. Material and Methods

### 2.1. Strains and Growth Conditions

Plasmid construction and experiments were performed in *E. coli* TOP10 cells (Invitrogen, Carlsbad, CA, USA). All products were purchased from Sigma-Aldrich (Sigma Aldrich BVBA, Overijse, Belgium) unless otherwise stated. Lysogeny Broth (LB) composed of 10 g/L tryptone (Becton, Dickinson and company, Erembodegem, Belgium), 5 g/L yeast extract (Becton) and 5 g/L NaCl was used to grow strains for routine cloning at 30 °C with shaking. LB agar plates of 12 g/L agar were used for single colony plating. All plasmids used in this study contained kanamycin resistance markers; thus, media were supplemented with 50 µg/mL kanamycin for selection. LB media were autoclaved before use. Experiments were performed in MOPS EZ Rich Defined Medium (Teknova, Hollister, CA, USA) with 2% glucose as the carbon source. The following chemical inducers were added to the medium: N-(3-oxododecanoyl)-L-homoserine lactone (O9139), N-(β-ketocaproyl)-L-homoserine lactone (K3007), chlorohydroquinone (technical grade, 85% purity) (224081), L-homocysteine (≥98.0% purity) (69453), calcium lactate heptahydrate from VWR (5551), vanillic acid with purity ≥97.0% (HPLC) (94770), copper(II) sulfate pentahydrate 98% (209198) and zinc sulfate heptahydrate from VWR (1884). A detailed description of inducer preparation and storage is given in Supplementary Materials 2. MOPS EZ Rich Defined Medium components and chemical inducers were solubilized in milli-Q water (mQ), with the exception of the former two chemical inducers, which were dissolved in DMSO, and of vanillic acid dissolved in EtOH. The ethanol was evaporated from the wells prior to additions of the growth medium. All components dissolved in mQ were filter-sterilized using syringe filters with a pore size of 0.2 µm (VWR International BVBA, Leuven, Belgium) before use.

### 2.2. Plasmid Construction

All plasmids were constructed using Golden Gate, as described in Section 2.1, using PaqCI and BsaI type II restriction enzymes (Bioké, Leiden, The Netherlands) according to the provider’s protocols. RBS libraries were generated using CPEC [25] with a degenerate sequence and DNA oligonucleotides purchased from IDT (Leuven, Belgium) and can be found in Supplementary Table S2. Construct verification was done using sequencing services (Macrogen Inc., Amsterdam, The Netherlands). The biosensor plasmids are medium-copy vectors with a broad-host replication of origin (pBBR1-MCS2) [26] and a kanamycin resistance marker. Supplementary Table S1 lists all plasmids used in this work. The annotated plasmid map of the MoBioS platform can be found in Supplementary Figure S4.

### 2.3. In Vivo Fluorescence Experiments

Prior to analysis, 4 biological replicates of each strain were inoculated in wells of 96-well microtiter plates (Greiner Bio-One bvba, Vilvoorde, Belgium) containing 150 µL LB with antibiotics and grown overnight on a Compact Digital Microplate shaker (Thermo Scientific) at 800 rpm and 30 °C. These cultures were diluted 1:300 in a final volume of 150 µL MOPS EZ Rich Defined Medium containing antibiotics and chemical inducers and grown in our Inheco Incubator Shaker MP (integrated in our Explorer G3 workstation, PerkinElmer, Waltham, MA, USA) at 30 °C and 800 rpm for 24 h. The strains were measured every 30 min for optical density at a wavelength of 600 nm and using mKate2 fluorescence with excitation and emission wavelengths set at 588 nm and 633 nm, respectively, in our PerkinElmer Ensight multimode plate reader (integrated in our explorer G3 workstation). TritonX-100, which was dissolved at 0.05 V% in 20 V% ethanol, was used to coat the corresponding plate lids to prevent condensation.

The SynMetR RBS library was analyzed with a similar set-up as described previously. A total of 76 RBS library colonies as singlets were grown prior to analysis, after which the library variants were diluted 1:300 in two plates containing either no ligands or 0.1 mM L-homocysteine. The control strains without MetR-related plasmids were grown in 4 biological replicates and further diluted in medium containing no ligands. All medium preparations and measurements were conducted as described for the ligand induction assay.

### 2.4. Data Processing and Statistical Analysis

The fluorescence output signal (*Fluo*), normalized for optical density (*OD*), was calculated as follows:

$${\left(\frac{Fluo}{OD_{600}}\right)}_{cor}\;=\;\frac{Fluo-Fluo_{med}}{OD_{600}-OD_{600,med}}-\frac{Fluo_{blank}-Fluo_{med}}{OD_{600,blank}-OD_{600,\;\;med}}$$

To correct the measurements for the background fluorescence and optical density of the medium, represented as *Fluo_med_* and *OD_600,med_* in the preceding equation, respectively, they were measured from wells containing MOPS EZ Rich Defined Medium without cell inoculation. The background fluorescence of the host was taken into consideration by measuring the fluorescence of SynJunk, an *E. coli* Top10 strain containing the MoBioS platform with non-functional DNA parts (*Fluo_blank_*), and normalizing for its optical density (*OD_600,blank_)*. Inter-plate variability caused by technical variation was accounted for by comparing the output of a reference culture containing the MoBioS platform with a constitutive promoter controlling mKate2 production. The mean of the corrected *Fluo/OD_600_* over the 4 biological replicates was fitted with the Hill function as described in [27].

## 3. Results

### 3.1. Modular Biological Sensor Platform: Plasmid Architecture Overview

An overview of the Modular Biological Sensor (MoBioS) platform and the corresponding biosensor construction step(s) is depicted in Figure 2. An annotated plasmid map of the MoBioS platform can be found in Supplementary Figure S4. Additionally, the completely annotated DNA sequence of the MoBioS platform plasmid is available at the Belgian Coordinated Collection of Microorganisms (BCCM), BCCM/Gene Corner in Ghent, Belgium. The plasmid can be ordered from the catalog under accession number LMBP 13776 (http://bccm.belspo.be/, accessed on 21 April 2023). The platform architecture is designed to allow a one-step, one-pot Golden Gate (GG) biosensor construction in which three DNA components are combined: (1) the MoBioS platform plasmid containing two cloning sites for insertion of biosensor-specific parts, (2) the transcription factor coding sequence (TF) of choice, including a synthetic RBS sequence, and (3) the corresponding responsive promoter with its transcription factor binding sites (PTFBS) of choice. All three elements have PaqCI GG restriction sites to enable the insertion of these parts into the platform. This assembly results in a synthetic biosensor architecture, here called SynSens (Figure 2), which consists of two independent operons, one for the detector module and one for the effector module (Figure 1). The TF of choice is constitutively expressed in the first operon, the detector module, by a synthetic promoter [28] and custom MoBioS-dedicated RBS sequence called the GoldenRBS. The second operon, the effector module, is responsible for the ligand-dependent biosensor output, and it consists of a fluorescent protein that is transcribed by the PTFBS promoter region of choice. The fluorescent protein mKate2 was chosen because of its limited interference with the autofluorescence of *E. coli* and for its fast maturation time [29]. Other transcriptional outputs, such as β-galactosidase, RNA aptamer–fluorophore systems, luminescent proteins, or bio-electrical outputs, can be integrated into the MoBioS platform, if desired. The specific architecture of the MoBioS platform ensures that the detector and effector modules are independent of each other and can, therefore, be engineered independently. Further, two specifically designed, non-coding DNA parts, here called junk parts, can be incorporated into the two modules in the same way as the biosensor parts, resulting in synthetic constructs with only a TF or PTFBS, which are utilized for benchmarking the biosensors. The annotated sequences for these two parts are listed in Supplementary Table S4. This platform comprises a customized medium-copy, broad-host plasmid backbone (based on pBBR1) and a specifically designed, GG-ready architecture. Use of the GG cloning technique allows the PCR-free assembly of standardized parts and plasmids [30].

In natural transcriptional regulatory circuits, the TF coding sequence is often found co-localized with a member of its regulon. In such cases, the TF and PTFBS region can be positioned in a flanking, bidirectional manner in which the intergenic PTFBS region also holds the promoter and RBS sequences for controlling the TF’s expression [31,32]. This can result in autoregulation of the TF’s expression level [33]. To avoid unwanted and unexpected regulatory effects from this bidirectional promoter on the TF expression level in the SynSens biosensors, a spacer and terminator sequence were added between the TF and mKate2 operons. In addition to enhancing the predictability of the response, the complete independence of the two modules facilitates further forward engineering of one module without interfering with the other.

However, constructing and characterizing the natural architecture of such a regulatory circuit can be of great interest for gathering fundamental knowledge. In addition, the bidirectional nature of the promoter region could be used as an additional biosensor tuning method by re-introducing this autoregulation of the TF, thereby mimicking the gene architecture of cis-acting TFs. To allow easy construction of these ‘natural’ gene architecture biosensors, here called NatSens, a novel feature was developed within the MoBioS system, namely the GoldenRBS. This synthetic RBS sequence is included in the TF part and regulates the translation initiation rate of the TF coding sequence. The GoldenRBS contains a BsaI GG site, which results in the restriction of the DNA exactly at the start of the CDS. A second BsaI GG site was introduced in the PTFBS part outside of the biologically relevant sequences, and it results in a four-base pair overhang that overlaps with the start codon of the CDS. When performing a GG reaction on the pSynSens biosensor plasmid, the two GG sites are cleaved, and the TF is linked to its original promoter-RBS region in a scarless manner, thereby generating the pNatSens biosensor plasmid (Figure 2). This allows for an accurate recreation of the natural gene architecture and re-establishes the natural autoregulation mechanism.

### 3.2. SynSens: Construction and Characterization of Biosensors with an Easily Tunable Synthetic Architecture

To demonstrate fast and efficient assembly through the MoBioS platform, the developed workflow was applied to eight different transcription factors (TFs) and corresponding responsive promoters. Furthermore, the TFs originated from four different TF families to establish the platform’s applicability across a wide range of the bacterial regulatory landscape (Supplementary Material 2). Two TFs from each family were selected together with a promoter containing the appropriate transcription factor binding site (PTFBS). Subsequently, the synthetic biosensors were constructed through a single GG assembly step, resulting in the high-throughput development of eight biosensors. *E. coli* strains were transformed with the resulting eight pSynSens plasmids. We refer to these strains by ‘Syn’, followed by the name of the transcription factor, e.g., SynLasR for the biosensor with transcription factor LasR.

**Figure 3:**
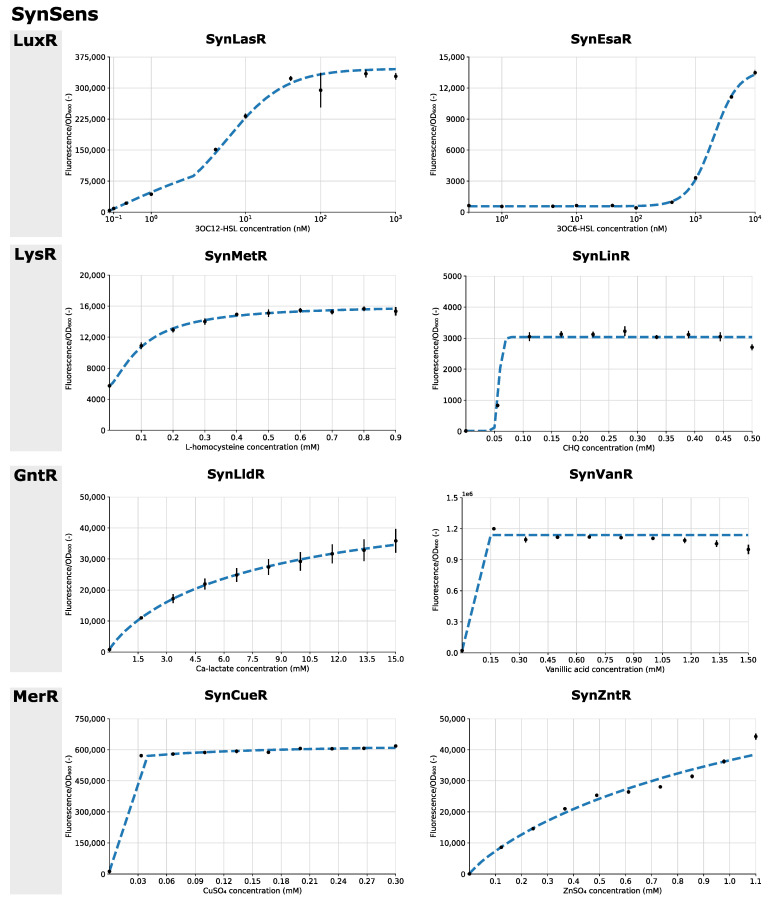
The response curves (black dots) and fitted Hill response curve (blue dashed line) of the eight biosensors over varying ligand concentration ranges after 24 h of growth are shown, grouped here by their corresponding transcription factor family (LuxR, LysR, GntR and MerR). For SynVanR only, the response is shown after 20 h of growth. Hill parameters are listed in Supplementary Material 2. Fluorescence is corrected for background fluorescence and normalized for optical density. Error bars depict standard errors over four biological replicates (*n* = 4). OD_600_ = optical density at 600 nm.

The functionality of the eight biosensors was tested in vivo via ligand response studies, with fluorescence as a proxy for transcriptional upregulation by the TF. Figure 3 summarizes the results of the fluorescent response to eight different ligand concentrations, which were chosen from the literature or from prior experiments, after 24 h of growth. From this data, response curves were obtained for all TFs. For the biosensors SynCueR and SynVanR, the lowest ligand concentration tested resulted in maximal induction of the responsive promoter. Nevertheless, this experiment demonstrates the functionality of these biosensors. As the supplementation of ligand molecules to the growth medium can influence the growth of the biosensor strains, the growth curves are provided in Supplementary Figure S1. Additionally, the fluorescent output of the biosensor strains can be found in Supplementary Figure S2. For the SynVanR sensor, a stationary growth phase was not observed after 24 h, which was potentially due to vanillic acid acting as an additional carbon source (Supplementary Figure S1). Therefore, the datapoints for obtaining the response curve for this biosensor were chosen after 20 h of growth instead of 24 h to ensure that the *OD_600_* values were still below 1 and, thus, linearly correlated with the number of cells. The growth of SynLinR was influenced by the level of chlorohydroquinone concentration, resulting in lag phases of differing lengths (Supplementary Figure S1).

For improving the interpretability and comparability of the response curves, Hill functions were fitted. This allows the estimation of relevant parameters, such as the maximal fluorescent signal and the TF–ligand affinity. It can be noted that the maximal fluorescent signal differed greatly between the different biosensors, as well as the fold change between the uninduced and induced stages. These parameters are intrinsic to the specific TF-PTFBS combination in use. All relevant biosensor information, such as the response curves, Hill parameters, DNA sequences and ligand information, is summarized in the biosensor ID sheets in Supplementary Material 2.

### 3.3. NatSens: Construction and Characterization of Biosensors in Their Natural Architecture

**Figure 4:**
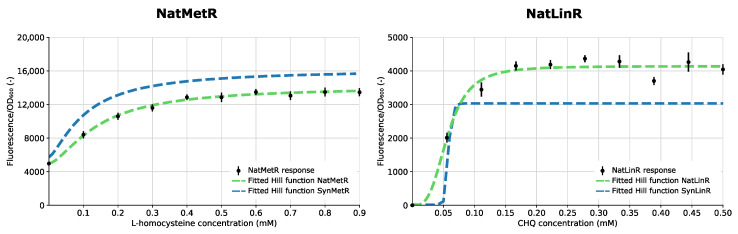
The response curves (black dots) and fitted Hill response curve (green dashed line) are plotted for the natural LysR-type transcriptional regulator biosensors, NatMetR and NatLinR, over varying concentration ranges after 24 h of growth. The fitted Hill function of the respective synthetic biosensor (blue striped line) is plotted as a reference. Hill parameters are listed in Supplementary Material 2. Fluorescence was corrected for background fluorescence and normalized for the optical density. Error bars depict standard errors over four biological replicates (*n* = 4). OD_600_ = optical density at 600 nm.

In nature, many of the regulators within the LysR-type transcriptional regulator (LTTR) family show co-localization with a gene of their regulon [34]. Therefore, the LTTR regulators described previously were chosen for the creation of the NatSens biosensors. These biosensors are referred to as ‘Nat’ followed by the transcription factor name, e.g., NatMetR. Using the built-in GoldenRBS, the ‘natural’ sensor constructs were created in parallel via a one-step BsaI GG reaction starting from their respective synthetic variants (Figure 2). This resulted in the positioning of the (naturally bidirectional) PTFBS directly upstream of the TF coding sequence. Figure 4 depicts the response curve and fitted Hill function of the NatSens constructs together with the fitted Hill function of their respective SynSens constructs. All relevant biosensor information is summarized in the biosensor ID sheets in Supplementary Material 2.

Differences in response curves between the synthetic (SynSens) and natural (NatSens) architecture for any biosensor can be attributed to either the re-establishment of any naturally present autoregulatory mechanism or the difference in TF expression levels caused by the change in the promoter–RBS combination that controls this expression level.

### 3.4. GoldenRBS Library for Fine-Tuning of Biosensor Response Curve

**Figure 5:**
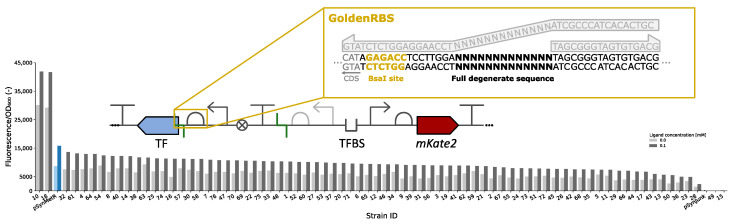
A schematic overview of the GoldenRBS library and the primers used to create the degenerate GoldenRBS sequence is depicted (top). The BsaI recognition sequence is highlighted in yellow. The fluorescence response, normalized for optical density, of 76 SynMetR GoldenRBS variants to 0 mM (light gray) and 0.1 mM (dark gray) L-homocysteine after 24 h of growth is plotted. The response of SynMetR is shown in light and dark blue as a reference. Fluorescence was corrected for background fluorescence and normalized for optical density. TF = transcription factor, TFBS = transcription factor binding site, CDS = coding sequence, OD_600_ = optical density at 600 nm.

The transcriptional regulatory mechanisms used for the development of biosensors have evolved in nature for specific metabolic or environmental sensing purposes. As such, the response curves are fine-tuned for their specific functions in vivo and do not necessarily meet the sensor characteristics required for specific applications. In addition to allowing fast and efficient conversion of the synthetic biosensor architecture to its original natural architecture (NatSens), the GoldenRBS is designed to be easily changed in a designated region without losing its GG functionality. This allows efficient and customized RBS strength tuning, modulation of TF expression levels and, thus, response curve characteristics. The GoldenRBS can be divided into three regions: two invariable flanking regions (one containing the GG site) and one variable region of 13 nucleotides that can be replaced by any specific or degenerate sequence of choice (Figure 5). Two designated GoldenRBS primers were designed to bind to the two invariable regions and to allow for the 5′ overhangs of these primers to be chosen freely (Supplementary Table S2). Using a PCR-based assembly technique such as CPEC [25], any desired 13-nucleotide sequence can be introduced to create either a (degenerate) library of RBS sequences or a single RBS sequence of choice. Because none of the primers rely on any of the sequences surrounding the RBS, such as the CDS of the TF, these primers can be used for any biosensor constructed with the MoBioS platform.

To demonstrate this feature, a GoldenRBS library was created for the developed SynMetR biosensor (Figure 5), allowing generation of new biosensor variants with different response curve characteristics. The 5′ overhangs of the two designated GoldenRBS primers were adapted to introduce a full degenerate 13-nucleotide sequence, generating a theoretical library size of 67 × 10^6^. Seventy-six variants from the obtained library were screened for their fluorescent response both in the absence and presence of the ligand (Figure 5). The optical densities at the time of measurement for all variants are provided in Supplementary Figure S3. A range of fluorescence intensities and fold changes between the induced and uninduced state were observed. In this initial screening, variants 10 and 18 demonstrated higher fluorescence/OD_600_ intensities compared to the original SynMetR. The other screened variants showed lower intensities.

## 4. Discussion

The numerous publications on the principles, applications and engineering possibilities of transcriptional biosensors indicate that this technology is a hot topic in the field of biotechnology [8,35,36,37]. Nevertheless, there is still a lack of standardized construction and characterization of these biosensors, which is essential for the expansion of the available portfolio in an efficient and high-throughput manner. Therefore, the MoBioS platform was designed to allow construction of a biosensor consisting of any transcription factor (TF) and promoter region (PTFBS) in a single Golden Gate (GG) reaction. Many cloning systems aiming for standardized construction, such as MoClo and GoldenBraid, have seen the strength of GG with its scarless, modular, one-pot nature [38,39]. It allows for the construction of a range of different plasmids according to strict design rules, resulting in standardized DNA parts and plasmids. However, the use of the MoClo and GoldenBraid systems for biosensor construction does not assure the reproducibility of the response curves across research groups, as the choices of all of the plasmid elements can influence the result. With the MoBioS platform, only the transcription factor and respective promoter differ across biosensors, thereby ensuring minimal differences between biosensor constructs.

The broad and easy applicability of the MoBioS platform was demonstrated with the construction of eight synthetic biosensors originating from four different transcription factor families. All eight biosensors showed significant responses to the addition of their respective ligands (Figure 3). For two of the biosensors, SynCueR and SynVanR, which detected CuSO_4_ and vanillic acid, respectively, the addition of the lowest tested ligand concentration activated these biosensors to their maximal output level. Additional experiments testing a new range of ligand concentrations could aid in further characterizing the response curves of these two biosensors in more detail. Furthermore, an influence of the added ligand on biosensor growth was observed for SynLinR and SynVanR (Supplementary Figure S1). The lag phase of SynLinR elongated with increasing chlorohydroquinone concentrations. This is due to the toxicity of this molecule for bacterial cells [40]. The ligand of SynVanR had the opposite effect and might have served as a carbon source. Therefore, growth continued after 20 h, and the stationary phase was not reached in the 24 h time interval that was monitored.

In addition to allowing the rapid and standardized construction of the desired transcriptional biosensor, the modular principle of the MoBioS platform also allows creation of the necessary control plasmids, which only contain the promoter or transcription factor. To this end, two non-coding DNA parts, here called junk parts, were designed for the MoBioS plasmid platform, enabling the creation of biosensor plasmids variants that only contain the TF part or PTFBS part (Supplementary Table S4). As such, the promoter activity in the absence of the TF can be studied. This is especially interesting for LysR-type transcriptional regulators, as these act as repressors in the absence and activators in the presence of their ligand [33,41]. By comparing the construct without TF with the constructs containing the TF, this repressor effect can be verified. Additionally, the platform provides the perfect framework for investigating the orthogonality of TFs and PTFBSs from different biosensors because these two elements are independently added to the Golden Gate reaction and can be any natural or non-natural pairing of TF and PTFBS parts.

It is important to note that response curves are construct- and host-dependent. Therefore, the MoBioS platform aims to increase the standardized use of these biosensors across labs to further improve the reusability and repeatability of results. In addition, all of the relevant biosensor information such as the response curves, Hill parameters, DNA sequences and information on the ligands were combined in ID sheets for each biosensor, which are provided in Supplementary Material 2. The ID sheets, together with the now-standardized biosensor layout, enhance the replicability of the results and allow for the creation of a biosensor database across labs. Currently, there are multiple databases collecting data on ligand-inducible transcription factors, TF–TFBS pairs or full-fledged biosensors. However, the gathered information differs greatly. GroovDB, the most recent biosensor database, provides information about biosensor sequences, ligand structures, genetic context and references to other databases (e.g., UniProt) and articles in the literature [42]. Additionally, all entries are required to have experimental evidence obtained with certain predefined techniques. However, the information requirements and novelty of this database cause it to be limited in the number of entries (101 at time of manuscript submission). In addition to GroovDB, other databases exist, such as RegulonDB, DBTBS, RegPrecise and PRODORIC [43,44,45,46]. In general, these databases lack information about the characteristics of the biosensor response curve. This is why our standardized MoBioS platform and biosensor ID sheets could further contribute to such databases and promote the widespread use of biosensors by providing a way to obtain replicable response curves.

In their natural genetic environment, transcriptional regulators are often co-localized with a gene of their regulon. This allows for efficient control due to spatial advantages, as the expressed protein can be easily recruited to its target promoter [31,47]. Often, the expression systems of both the regulator and its target gene overlap, which can enable the regulator to co-regulate its own expression, i.e., autoregulation. This creates an extra layer of regulation, which can be desired or undesired depending on the biosensor application [31,32]. To allow easy and high-throughput creation of this natural architecture, the MoBioS platform incorporates GoldenRBS to convert the pSynSens plasmid into the pNatSens plasmid through a one-pot, one-step GG reaction (Figure 2). The resulting NatSens biosensors can provide insights into naturally present mechanisms, e.g., autoregulation, and thereby aid in gaining fundamental knowledge on such regulatory circuits. In the current study, the natural biosensor variants of both SynLinR and SynMetR (NatLinR and NatMetR, respectively) were constructed as proofs of concept. NatMetR showed a lower maximal response than SynMetR, yet the sensors behaved similarly over the given range of ligands (Figure 4). The lower output can be linked to the previously reported negative autoregulation [48,49], which causes a reduction in regulator concentration. For the LinR biosensors, the difference between the response curves was bigger than that observed for the MetR biosensors. The SynLinR regulator showed a steeper increase at the lower concentrations, indicating that its response to chlorohydroquinone (CHQ) will be linear over a narrower range of ligand concentrations. The NatLinR response seems to increase more gradually, which is also indicated by the higher Hill coefficient (n). Any differences in the response curves between SynSens and NatSens biosensor architectures can arise from the re-establishment of any naturally present autoregulatory mechanisms. In addition, the switch from the synthetic promoter–GoldenRBS combination to the natural promoter–RBS combination will give rise to different TF expressions and potentially alter the response curve. As demonstrated by the RBS library in Figure 5, the TF expression level can greatly influence the level of fluorescence [50].

In addition to allowing the easy construction of the natural architecture of the biosensors, GoldenRBS provides a way to vary the expression level of the transcription factor in a construct independent manner. With simple primer design and a single CPEC reaction, a single GoldenRBS variant or library of GoldenRBS variants can be created. The transcription factor expression level is used as a tuning parameter to influence many aspects of the response curve, such as leaky expression and maximal fluorescence. The potential of this GoldenRBS was demonstrated by creating a full degenerate RBS library of the SynMetR biosensor. Thirteen nucleotides were varied, leading to a library size of 67 × 10^6^. From this library, a small fraction (76 variants) was screened. The fluorescence intensity was measured in the absence and presence of the ligand (Figure 5). To screen for a possible shift in the response curve, a concentration of 0.1 mM L-homocysteine was added to the medium. In our earlier characterization assay, this concentration corresponded to an intermediate response from the biosensor. Therefore, a lower or higher response from a variant might indicate a horizontal shift of this response curve to higher or lower concentrations, respectively. In Figure 5, two of the screened variants showed higher fluorescence intensity in the presence of the ligand compared to the original SynMetR biosensor. However, the uninduced state reaches higher values as well. Therefore, it is unclear if a complete horizontal shift of the response curve was obtained, or if it was solely an increase in maximal promoter activity. Nonetheless, it was demonstrated that varying the TF expression level can be used to tune the output of the biosensor. By combining degenerate libraries with high-throughput techniques, such as FACS, larger screenings can be performed to find the desired output.

## 5. Conclusions

The MoBioS platform allows for fast, efficient and standardized biosensor construction, as demonstrated by the parallel construction of eight biosensors. In addition, the work presented here shows that regulators from different protein families were functional in the new genetic context, thereby demonstrating the use of the platform for regulators over a large part of the transcription regulator landscape. The resulting biosensors were subsequently characterized within the standardized backbone and extensively described, which should improve their application in different contexts and facilitate replication of the results by different research groups. In addition to standardized biosensor creation, we also highly advise the communication of (meta)data for future biosensor constructs.

The platform also includes several ways to alter the biosensors’ characteristics. First, because of the independent detector and effector module in the SynSens architecture, engineering of the TFBS, RBS or promoter in the effector module will not compromise the expression of the transcription factor in the detector module, and vice versa. Therefore, PTFBS parts and/or TF parts can consist of libraries of sequence variants to construct libraries of biosensor variants in a high-throughput, sequence-independent manner using the implemented Golden Gate assembly. Second, as the concentration of the regulator has a great impact on the response curve, the GoldenRBS was designed to be easily altered in strength by simple PCR based techniques. Last, the incorporation of a GG site in the GoldenRBS, allows for a one-step, scarless recreation of the natural gene architecture for gaining more fundamental knowledge.

## Data Availability

Data is available upon request.

## Supplementary Materials

The following supporting information can be downloaded at: https://www.mdpi.com/article/10.3390/bios13060590/s1, containing: Figure S1: Growth curves of the biosensors with different ligand concentrations. Figure S2: Fluorescence curves of the biosensors with different ligand concentrations. Figure S3: Optical density of the MetR RBS library. Figure S4: Annotated plasmid map of the MoBioS platform. Table S1: List of the plasmids used and constructed in this study. Table S2: List of primer sequences used for generating the GoldenRBS library. Table S3: Annotated sequences of the MoBioS platform parts. Table S4: Annotated sequence of the junk DNA parts that can be inserted into the MoBioS platform. Supplementary Materials 2 contains the ID sheets of all of the created biosensors.

## References

[1] Saha B.C. (2003). Commodity Chemicals Production by Fermentation: An Overview. Fermentation Biotechnology.

[2] Petzold C.J., Chan LJ G., Nhan M., Adams P.D. (2015). Analytics for metabolic engineering. Front. Bioeng. Biotechnol..

[3] Kosuri S., Church G.M. (2014). Large-scale de novo DNA synthesis: Technologies and applications. Nat. Methods.

[4] Mahr R., Frunzke J. (2016). Transcription factor-based biosensors in biotechnology: Current state and future prospects. Appl. Microbiol. Biotechnol..

[5] Safaei M., Mobini G.R., Abiri A., Shojaeian A. (2020). Synthetic biology in various cellular and molecular fields: Applications, limitations, and perspective. Mol. Biol. Rep..

[6] Fernandez-López R., Ruiz R., de la Cruz F., Moncalián G. (2015). Transcription factor-based biosensors enlightened by the analyte. Front. Microbiol..

[7] Ma Z., Jacobsen F.E., Giedroc D.P. (2009). Metal Transporters and Metal Sensors: How Coordination Chemistry Controls Bacterial Metal Homeostasis. Chem. Rev..

[8] De Paepe B., Peters G., Coussement P., Maertens J., De Mey M. (2017). Tailor-made transcriptional biosensors for optimizing microbial cell factories. J. Ind. Microbiol. Biotechnol..

[9] Koch M., Pandi A., Borkowski O., Cardoso Batista A., Faulon J.L. (2019). Custom-made transcriptional biosensors for metabolic engineering. Curr. Opin. Biotechnol..

[10] Stewart A.J., Plotkin J.B. (2012). Why transcription factor binding sites are ten nucleotides long. Genetics.

[11] Schallmey M., Frunzke J., Eggeling L., Marienhagen J. (2014). Looking for the pick of the bunch: High-throughput screening of producing microorganisms with biosensors. Curr. Opin. Biotechnol..

[12] Kaczmarek J.A., Prather K.L.J. (2021). Effective use of biosensors for high-throughput library screening for metabolite production. J. Ind. Microbiol. Biotechnol..

[13] Boada Y., Vignoni A., Picó J., Carbonell P. (2020). Extended Metabolic Biosensor Design for Dynamic Pathway Regulation of Cell Factories. iScience.

[14] Chou H.H., Keasling J.D. (2013). Programming adaptive control to evolve increased metabolite production. Nat. Commun..

[15] Xia P.-F., Ling H., Foo J.L., Chang M.W. (2019). Synthetic genetic circuits for programmable biological functionalities. Biotechnol. Adv..

[16] Kim S.G., Noh M.H., Lim H.G., Jang S., Jang S., Koffas M.A.G., Jung G.Y. (2018). Molecular parts and genetic circuits for metabolic engineering of microorganisms. FEMS Microbiol. Lett..

[17] Min B.E., Hwang H.G., Lim H.G., Jung G.Y. (2017). Optimization of industrial microorganisms: Recent advances in synthetic dynamic regulators. J. Ind. Microbiol. Biotechnol..

[18] Smanski M.J., Zhou H., Claesen J., Shen B., Fischbach M.A., Voigt C.A. (2016). Synthetic biology to access and expand nature’s chemical diversity. Nat. Rev. Microbiol..

[19] Gao C., Xu P., Ye C., Chen X., Liu L. (2019). Genetic Circuit-Assisted Smart Microbial Engineering. Trends Microbiol..

[20] Jeon Y., Lee Y., Kim K., Jang G., Yoon Y. (2022). Transcription Factor-Based Biosensors for Detecting Pathogens. Biosensors.

[21] Guo M., Du R., Xie Z., He X., Huang K., Luo Y., Xu W. (2019). Using the promoters of MerR family proteins as “rheostats” to engineer whole-cell heavy metal biosensors with adjustable sensitivity. J. Biol. Eng..

[22] Gallup O., Ming H., Ellis T. (2021). Ten future challenges for synthetic biology. Eng. Biol..

[23] Müller K.M., Arndt K.M. (2012). Standardization in synthetic biology. Methods Mol. Biol..

[24] Decoene T., De Paepe B., Maertens J., Coussement P., Peters G., De Maeseneire S.L., De Mey M. (2017). Standardization in synthetic biology: An engineering discipline coming of age. Crit. Rev. Biotechnol..

[25] Quan J., Tian J. (2009). Circular polymerase extension cloning of complex gene libraries and pathways. PLoS ONE.

[26] Kovach M.E., Elzer P.H., Hill D.S., Robertson G.T., Farris M.A., Roop R.M., Peterson K.M. (1995). Four new derivatives of the broad-host-range cloning vector pBBR1MCS, carrying different antibiotic-resistance cassettes. Gene.

[27] De Paepe B., Maertens J., Vanholme B., De Mey M. (2018). Modularization and Response Curve Engineering of a Naringenin-Responsive Transcriptional Biosensor. ACS Synth. Biol..

[28] De Mey M., Maertens J., Lequeux G.J., Soetaert W.K., Vandamme E.J. (2007). Construction and model-based analysis of a promoter library for *E. coli*: An indispensable tool for metabolic engineering. BMC Biotechnol..

[29] Shcherbo D., Murphy C.S., Ermakova G.V., Solovieva E.A., Chepurnykh T.V., Shcheglov A.S., Verkhusha V., Pletnev V.Z., Hazelwood K.L., Roche P.M. (2009). Far-red fluorescent tags for protein imaging in living tissues. Biochem. J..

[30] Engler C., Kandzia R., Marillonnet S. (2008). A one pot, one step, precision cloning method with high throughput capability. PLoS ONE.

[31] Yamada M., Kabir S., Tsunedomi R. (2003). Divergent Promoter Organization May Be a Preferred Structure for Gene Control in *Escherichia coli*. J. Mol. Microbiol. Biotechnol..

[32] Beck C.F., Warren R.A. (1988). Divergent promoters, a common form of gene organization. Microbiol. Rev..

[33] Maddocks S.E., Oyston P.C.F. (2008). Structure and function of the LysR-type transcriptional regulator (LTTR) family proteins. Microbiology.

[34] Lindquist S., Lindberg F., Normark S. (1989). Binding of the Citrobacter freundii ampR regulator to a single DNA site provides both autoregulation and activation of the inducible ampC β-lactamase gene. J. Bacteriol..

[35] Ding N., Zhou S., Deng Y. (2021). Transcription-Factor-based Biosensor Engineering for Applications in Synthetic Biology. ACS Synth. Biol..

[36] Li C., Wang C., Zhu J., Xue F., Sun X., Gu Y. (2022). Advances and prospects of transcription-factor-based biosensors in high-throughput screening for cell factories construction. Food Bioeng..

[37] Mitchler M.M., Garcia J.M., Montero E.N., Williams G.J. (2021). Transcription factor-based biosensors: A molecular-guided approach for natural product engineering. Curr. Opin. Biotechnol..

[38] Weber E., Engler C., Gruetzner R., Werner S., Marillonnet S. (2011). A Modular Cloning System for Standardized Assembly of Multigene Constructs. PLoS ONE.

[39] Sarrion-Perdigones A., Falconi E.E., Zandalinas I.S., Juárez P., Fernández-Del-Carmen M.A., Granell A., Orzaez D. (2011). GoldenBraid: An Iterative Cloning System for Standardized Assembly of Reusable Genetic Modules. PLoS ONE.

[40] Murugesan K., Chang Y.-Y., Kim Y.-M., Jeon J.-R., Kim E.-J. (2010). Enhanced transformation of triclosan by laccase in the presence of redox mediators. Water Res..

[41] Picossi S., Belitsky B.R., Sonenshein A.L. (2007). Molecular Mechanism of the Regulation of Bacillus subtilis gltAB Expression by GltC. J. Mol. Biol..

[42] D’oelsnitz S., Love J.D., Diaz D.J., Ellington A.D. (2022). GroovDB: A Database of Ligand-Inducible Transcription Factors. ACS Synth. Biol..

[43] Santos-Zavaleta A., Salgado H., Gama-Castro S., Sánchez-Pérez M., Gómez-Romero L., Ledezma-Tejeida D., García-Sotelo J.S., Alquicira-Hernández K., Muñiz-Rascado L.J., Peña-Loredo P. (2019). RegulonDB v 10.5: Tackling challenges to unify classic and high throughput knowledge of gene regulation in *E. coli* K-12. Nucleic Acids Res..

[44] Sierro N., Makita Y., De hoon M., Nakai K. (2008). DBTBS: A database of transcriptional regulation in Bacillus subtilis containing upstream intergenic conservation information. Nucleic Acids Res..

[45] Novichkov P.S., Kazakov A.E., Ravcheev D.A., Leyn S.A., Kovaleva G.Y., Sutormin R.A., Kazanov M.D., Riehl W., Arkin A.P., Dubchak I. (2013). RegPrecise 3.0––A resource for genome-scale exploration of transcriptional regulation in bacteria. BMC Genomics.

[46] Dudek C.-A., Jahn D. (2021). PRODORIC: State-of-the-art database of prokaryotic gene regulation. Nucleic Acids Res..

[47] Wu K., Rao C.V. (2010). The role of configuration and coupling in autoregulatory gene circuits. Mol. Microbiol..

[48] Cai X.-Y., Redfield B., Maxon M., Weissbach H., Brot N. (1989). The effect of homocysteine on metR regulation of metE, metR and metH expression in vitro. Biochem. Biophys. Res. Commun..

[49] Wu W.F., Urbanowski M.L., Stauffer G.V. (1995). Characterization of a second MetR-binding site in the metE metR regulatory region of Salmonella typhimurium. J. Bacteriol..

[50] Glascock C.B., Weickert J.M. (1998). Using chromosomal lacIQ1 to control expression of genes on high-copy-number plasmids in *Escherichia coli*. Gene.

