# Supplementary material for: MoBioS: Modular Platform Technology for High-Throughput Construction and Characterization of Tunable Transcriptional Biological Sensors
Source: Biosensors (Basel). 2023 May 30;13(6):590. doi: 10.3390/bios13060590 (PMC10296306; doi:10.3390/bios13060590)
Supplement: Supplementary file 1 [file biosensors-13-00590-s001.zip › Supplementary Material 1.pdf]

Supplementary data 1

MoBioS: Modular platform technology for high-throughput construction and characterization of tunable transcriptional biological sensors

*Demeester W.[1], De Baets J.[1], De Mey M., De Paepe B. **
Centre for Synthetic Biology (CSB), Ghent university, 900 Ghent, Belgium
[1] = Equal contribution

* Corresponding author: Brecht.DePaepe@UGent.be

Overview

Supplementary Figure S1. Growth curves of the biosensors with different ligand concentrations.

Supplementary Figure S2. Fluorescence curves of the biosensors with different ligand concentrations.

Supplementary Figure S3. Optical density of the MetR RBS library.

Supplementary Figure S4. Annotated plasmid map of the MoBioS platform.

Supplementary Table S1. List of the plasmids used and constructed in this study.

Supplementary Table S2. List of primer sequences used for generating the GoldenRBS library.

Supplementary Table S3. Annotated sequences of the MoBioS platform parts.

Supplementary Table S4. Annotated sequence of the junk DNA-parts that can be inserted into the MoBioS platform.

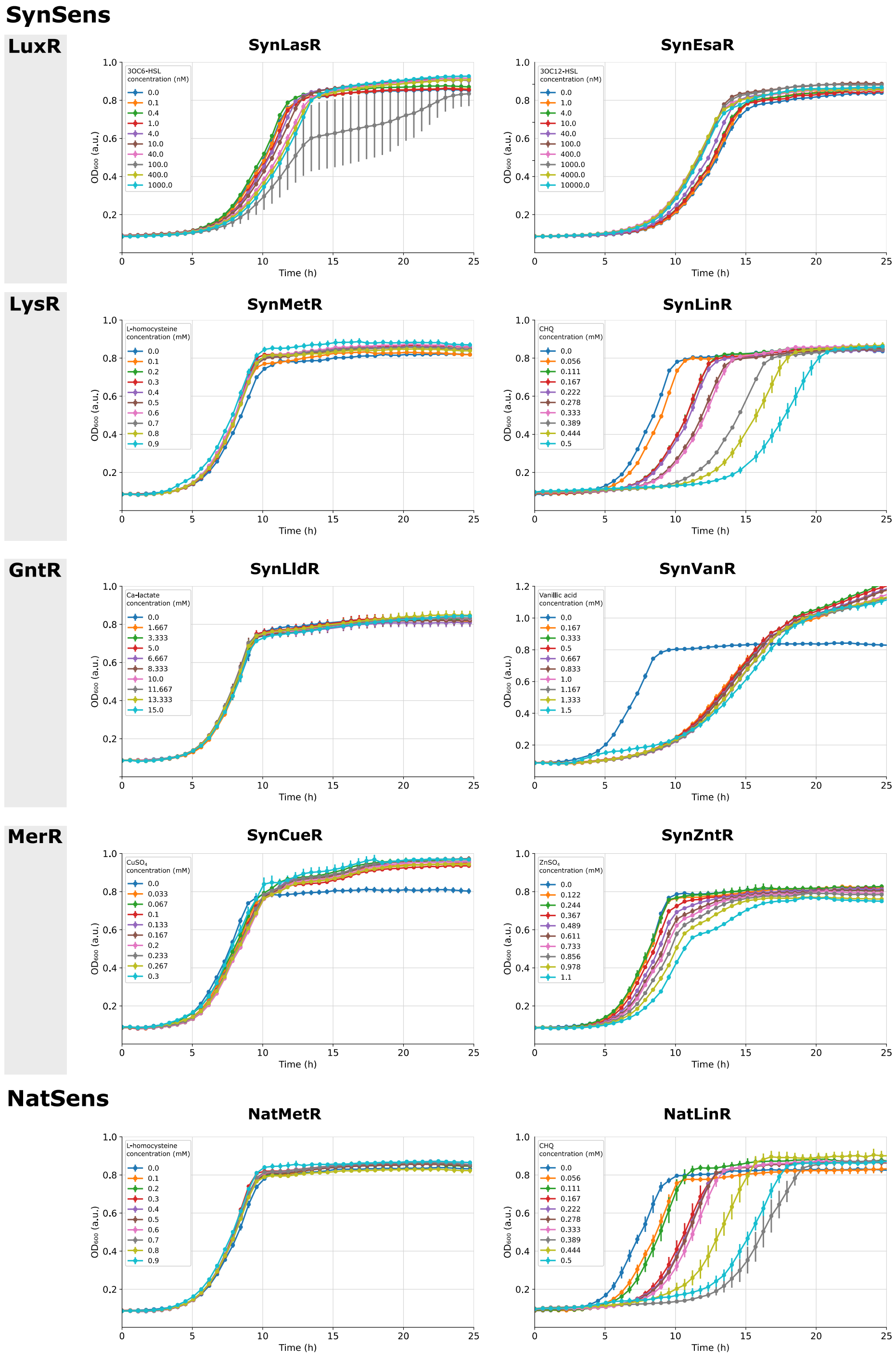

Supplementary Figure S1. This figure gathers the growth curves of the biosensor strains with the addition of different ligand concentrations. Error bars represent standard errors from the plotted mean value over four biological replicates. SynVanR plotted OD_{600} range plotted up to 1.2 due to higher growth. OD_{600} = optical density measured at 600 nm.

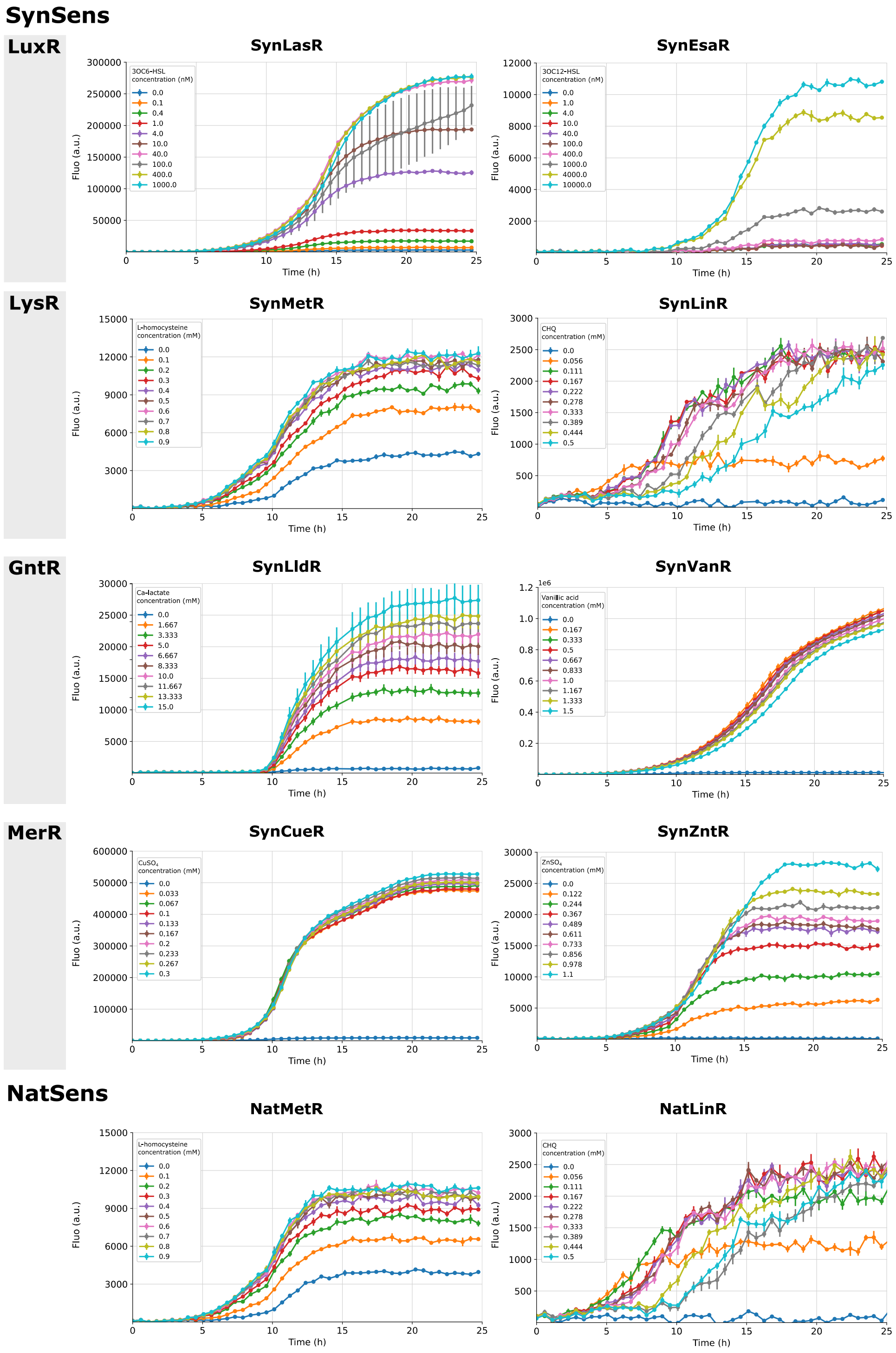

Supplementary Figure S2. This figure gathers the fluorescence of the biosensor strains with the addition of different ligand concentrations. Error bars represent standard errors from the plotted mean value over four biological replicates. Fluo = mKate2 fluorescence with excitation and emission wavelengths set at 588 nm and 633 nm, respectively.

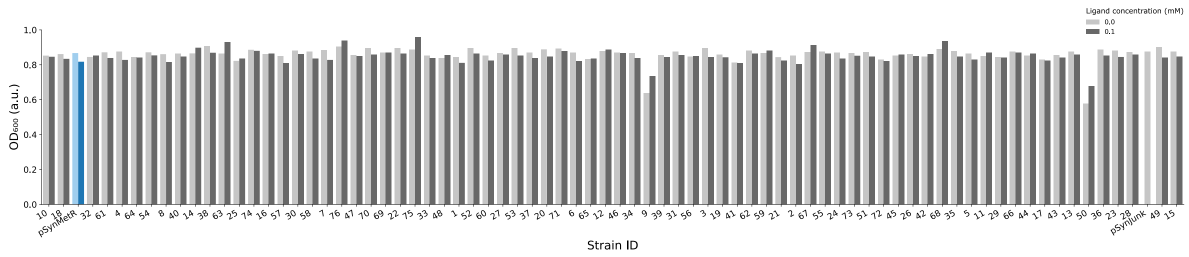

Supplementary Figure S3. This figure gathers the optical density of the MetR RBS library variants after 24h of incubation in both the uninduced (light gray) state and in the presence of 0.1 mM L-homocysteine (dark gray). Growth of the SynMetR is shown in light and dark blue as a reference. OD_{600} = optical density measured at 600 nm.

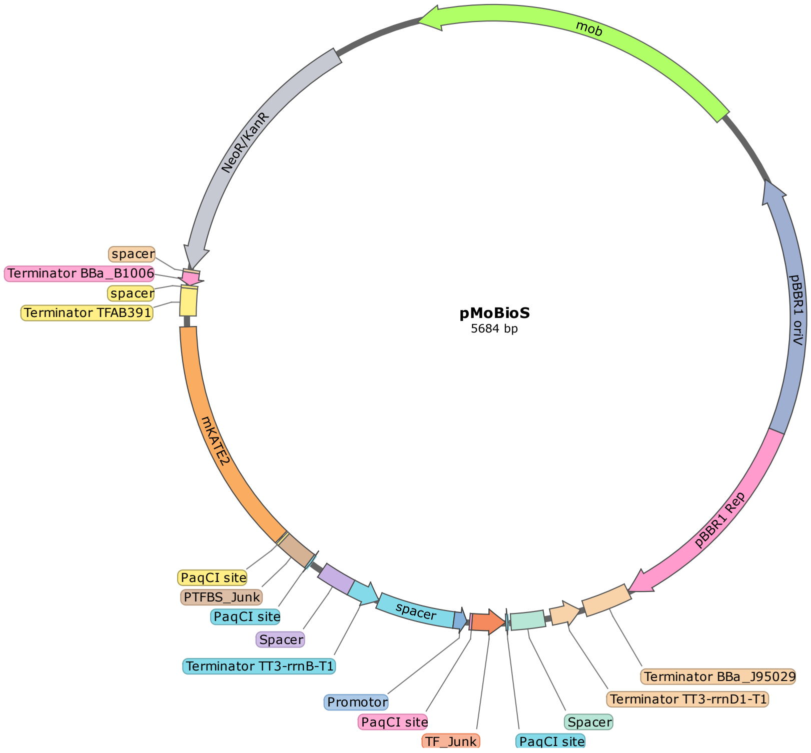

Supplementary Figure S4. The plasmid map of the MoBioS platform, generated with SnapGene, is shown. Two non-coding DNA regions surrounded by PaqCI Golden Gate sites will be replaced with the transcription factor (TF) and promoter part (PTFBS) after the Golden Gate reaction. The PTFBS will then be located directly upstream of the *mKate2* gene. The TF gene (with RBS) is inserted downstream of the P22 promoter.

Supplementary Table S1. An overview is given of the plasmids used in this research.

Strain	Transcription factor	Promoter region
**Platform**		
MoBioS	TF_junk	PTFBS_junk
**Synthetic architecture**		
SynLasR	LasR	P_{lasI}
SynEsaR	EsaR	P_{esaR}
SynLinR	LinR	P_{linER}
SynMetR	MetR	P_{metER}
SynLldR	LldR	P_{lldARD}
SynVanR	VanR	P_{vanCC}
SynCueR	CueR	P_{copA}
SynZntR	ZntR	P_{zntAR}
**Natural architecture**		
NatLinR	LinR	P_{linER}
NatMetR	MetR	P_{metER}

Supplementary Table S2. An overview is given of the primers used for the construction of the GoldenRBS library.

Reverse_GoldenRBS_library	CGTCACACTACCCGCTANNNNNNNNNNNNNTCCAAGGAGGTCTCTATG
Forward_GoldenRBS	TAGCGGGTAGTGTGACG

Supplementary Table S3. Annotated sequences of the MoBioS platform parts. TF = transcription factor PTFBS = promoter with transcription factor binding sites.

TF-part	GATGATGGCGAGCATTCACCTGCGGTAGCTANNNNNNNNNNNNNNNNNNNNNNNNNNN<br>PaqCI recognition sequence; PaqCI restriction site; Transcription factor gene<br>NNNNNNNNNNNNNNNNNNNNNNNNCATAGAGACCTCCTTGGACCTGGTACGTCGCTAGC<br>BsaI restriction site; BsaI recognition sequence; Golden RBS<br>GGGTAGTGTGACGTGGCGCAGGTGATGGACTTCATGCTGAC<br>PaqCI restriction site; PaqCI recognition sequence
PTFBS-part	GATGATGGCGAGCATTCACCTGCGGTACTTACGGTCTCTNCATNNNNNNNNNNNNNNNNN<br>PaqCI recognition sequence; PaqCI restriction site; BsaI recognition sequence; BsaI restriction site<br>NNNNNNNNNNNNNNNNNNNNNNNNNNNNNNNNNATGGTGGCGCAGGTGATGGACTTC<br>Promoter region; PaqCI restriction site; PaqCI recognition sequence<br>ATGCTGAC

Supplementary Table S4. Annotated sequence of the junk DNA-parts that can be inserted into the MoBioS platform.

TF-junk	GATGATGGCGAGCATTCACCTGCGGTAGCTAAGACGAATTACTTATCTGGCAGGAGTATCGT<br>PaqCI recognition sequence PaqCI restriction site<br>CCGTAGTTTCAACTGTTCTGTGCATACGGCCCTGAAAGACTATTGATTACGAATATAAGGAAA<br>ACCACTAGAGACCTCCTTGGACCTGGTACGTCGCTAGCGGGTAGTGTGACGTGGCGCAGGT<br>PaqCI restriction site PaqCI recognition sequence PaqCI restriction site PaqCI recognition sequence<br>GATGGACTTCATGCTGAC
PTFBS-junk	GATGATGGCGAGCATTCACCTGCGGTACTTACGGTCTCTCACTAAATGAACGATTTCTTAGTC<br>PaqCI recognition sequence PaqCI restriction site BsaI recognition sequence BsaI restriction site<br>GGCGTTATAGTAAGTCACTCTTTTTCAGCGGTATTTTAAAGATGAGAAAGCGATGGTCAAGC<br>GTGGTCTGCCTGAAGTCTATGGTGGCGCAGGTGATGGACTTCATGCTGAC<br>PaqCI restriction site PaqCI recognition sequence
