# Supplementary material for: MoBioS: Modular Platform Technology for High-Throughput Construction and Characterization of Tunable Transcriptional Biological Sensors
Source: Biosensors (Basel). 2023 May 30;13(6):590. doi: 10.3390/bios13060590 (PMC10296306; doi:10.3390/bios13060590)
Supplement: Supplementary file 1 [file biosensors-13-00590-s001.zip › Supplementary Material 2.pdf]

# Supplementary data 2

# MoBioS: Modular platform technology for high-throughput construction and characterization of tunable transcriptional biological sensors

*Demeester W.*[1]*, De Baets J.*[1]*, Duchi D., De Mey M., De Paepe B*.*
*Centre for Synthetic Biology (CSB), Ghent university, 900 Ghent, Belgium*
[1] = Equal contribution

* Corresponding author: Brecht.DePaepe@UGent.be

## Overview

ID-sheets of the biosensors constructed in this research. They contain information on the ligand, response curve, meta-data about the strain and experiment and the DNA-sequences of the transcription factor and promoter part.

# LasR:3OC12-HSL

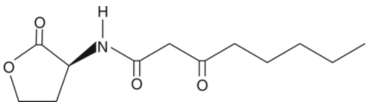


Ligand: 3-oxo-dodecanoyl-L-homoserine lactone
Source: Sigma-Aldrich O9139
CAS ID: 168982-69-2
Stock: 1 mM
Solvent: DMSO
Storage: -80 °C (months)

## Response curve: SynLasR

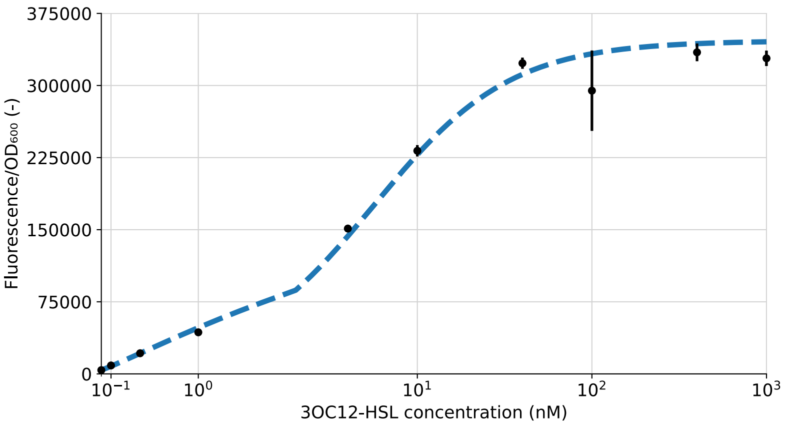


### Hill function

$$\left(\frac{\overline{\text{Fluo}}}{\text{OD}_{600}}\right)_{cor} = f(C) = a + k\left(\frac{C^n}{C^n + K^n}\right)$$

### Parameters Hill function

$a_{(a.u)} = 4.09*10^3 \pm 0.11*10^3$
$k_{(a.u)} = 3.42*10^5 \pm 0.05*10^5$
$M_{(a.u)} = 3.47*10^5 \pm 0.05*10^5$
$n = 1.11 \pm 0.02$
$K_{(nM)} = 5.63 \pm 0.23$

Error bars depict standard errors over four biological replicates (n=4)
a = the basal normalized fluorescent signal (leaky expression, a.u.)
k = the maximum normalized fluorescent signal relative to a (a.u.)
M = a + k = the maximum normalized fluorescent signal (a.u.)
n = the Hill coefficient (cooperativity, sigmoid character)
K = the Hill constant (TF-ligand affinity, nM)
C = the ligand concentration in the growth medium (nM)

## Metadata

| | |
|---|---|
| Host | *Escherichia coli TOP10 (F- mcrA Δ(mrr-hsdRMS-mcrBC) Φ80lacZΔM15 ΔlacX74 recA1 araD139 Δ(araleu)7697 galU galK rpsL (StrR) endA1 nupG)* |
| Origin | *Pseudomonas aeruginosa* PAO1 |
| Parts | LasR: Gene ID: 881789 codon optimized for *Escherichia coli* |
| | $P_{LasI}$: Gene ID: 881777, promoter upstream of *lasI* |
| Architecture | Synthetic |
| Experiment | 150 µL EZ Rich medium, 96-well black microtiter plate<br>PerkinElmer Ensight, exc. 588nm, emm. 633nm at 30°C<br>Fluorescence after 24h of growth |
| Notes | / |

# LasR:3OC12-HSL

| | |
|---|---|
| *lasR* | GATGATGGCGAGCATTCACCTGCGGTAGCTA**TTACAGGGTAATCAGACCCAGATTAACT**<br>PaqCI recognition sequence PaqCI restriction site<br>**GCCATAATTGCTGCAACACGACGGCTGGTAACACCAAATTTGCGACGAATATTACCC**<br>**ATATGAAAGTTCACGTTGGCTTCGCTACAATTACAAATAACGCTAATTTCCCAGCTG**<br>**GTTTTACCAATTGCACACCACTGCAGAACTTCTTTTTCACGGCTGGTCAGAACCACC**<br>**GGTTTGCTAACCGGATGTTCAAATGCCAGACCGGCACCGCTCTGCAGTGCATAATCT**<br>**TTCAGCATCCACAGTGTCGGCAGCACGCTTTCCATAAAACGATTTGCTTCGGCACGA**<br>**TTTTCTGCTTCAACGCTCAGGCTCAGTGCACCCAGTTCACCACGTGCGCCATGCAGC**<br>*lasR*<br>**GGCATGGTCAGACCATAAACCAGACCTGCTGCGCTTGCTTCTTCAAAAAATTCGTGC**<br>**TGTTTACGGGTCTGATAAATGCTCGGTTCCCAAAAAATCGGCAGAACGCTCTGGGTA**<br>**CAATGGCTAACGGTCGGATCAACACGTGCATAACCTGCACGATCATAATGTTCACGC**<br>**CATGCTGCCGGATAATTACCCACGATAAAGGCATTTTCATAATCCTGGCTATCTTTC**<br>**GGCAGCAGACCAAACAGAATTTTGCTAAAACCCAGATCGCTTGCCATTTTCTGCAGG**<br>**ATTGCTGACCATTCCAGTTTACCGCTGCTACGTTCCAGTTCCAGAAAACCATCAACC**<br>**AGTGCCAT**AGAGACCTCCTTGGACCTGGTACGTCGCTAGCGGGTAGTGTGACGTGGCGCA<br>BsaI restriction site BsaI recognition sequence GoldenRBS PaqCI restriction site<br>GGTGATGGACTTCATGCTGAC<br>PaqCI recognition sequence |
| $P_{lasI}$ | GATGATGGCGAGCATTCACCTGCGGTACTTA**TTCGAGCCTAGCAAGGGTCCGGGTTCAC**<br>PaqCI recognition sequence PaqCI restriction site<br>**CGAAATCTATCTCATTTGCTAGTTATAAAATTATGAAATTTGCATAAATTCTTCA**TCT<br>$P_{LasI}$<br>AGAGATTAAAGAGGAGAAATACTAGATGGTGGCGCAGGTGATGGACTTCATGCTGAC<br>Bba_B0030 PaqCI restriction site PaqCI recognition sequence |

# EsaR:3OC6-HSL

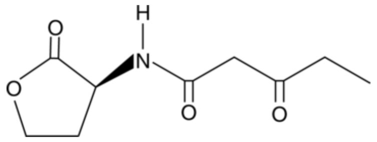


Ligand: 3-oxo-hexanoyl-L-homoserine lactone
Source: Sigma-Aldrich K3007
CAS ID: 143537-62-6
Stock: 1 mM
Solvent: DMSO
Storage: -80 °C (2 months)

## Response curve: SynEsaR

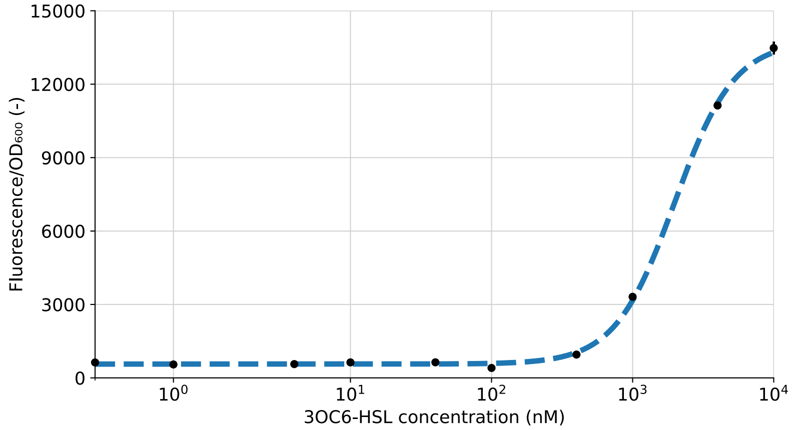


### Hill function

$$\left(\frac{\overline{\text{Fluo}}}{\text{OD}_{600}}\right)_{cor} = f\,(C) = a + k\left(\frac{C^n}{C^n + K^n}\right)$$

### Parameters Hill function

$a_{\,(a.u)} = 5.64*10^2 \pm 0.23*10^2$
$k_{\,(a.u)} = 1.32*10^4 \pm 0.04*10^4$
$M_{\,(a.u)} = 1.38*10^4 \pm 0.04*10^4$
$n = 2.05 \pm 0.10$
$K_{\,(nM)} = 1.98*10^3 \pm 0.11*10^3$

Error bars depict standard errors over four biological replicates (n=4)
a = the basal normalized fluorescent signal (leaky expression, a.u.)
k = the maximum normalized fluorescent signal relative to a (a.u.)
M = a + k = the maximum normalized fluorescent signal (a.u.)
n = the Hill coefficient (cooperativity, sigmoid character)
K = the Hill constant (TF-ligand affinity, nM)
C = the ligand concentration in the growth medium (nM)

## Metadata

| | |
|---|---|
| Host | *Escherichia coli TOP10 (F- mcrA Δ(mrr-hsdRMS-mcrBC) Φ80lacZΔM15 ΔlacX74 recA1 araD139 Δ(araleu)7697 galU galK rpsL (StrR) endA1 nupG)* |
| Origin | *Pantoea stewartii* |
| Parts | EsaR: GenBank L32184.1 partly codon-optimized for *Escherichia coli* |
| | $P_{esaR}$: GenBank L32184.1 |
| Architecture | Synthetic |
| Experiment | 150 µL EZ Rich medium, 96-well black microtiter plate<br>PerkinElmer Ensight, exc. 588nm, emm. 633nm at 30°C<br>Fluorescence after 24h of growth |
| Notes | EsaR works as a repressor of $P_{esaR}$ in the absence of the ligand. The promoter region is bidirectional and EsaR activates $P_{esaS}$ in the absence of the ligand. |

# EsaR:3OC6-HSL

| | |
|---|---|
| *esaR* | GATGATGGCGAGCATTCACCTGCGGTAGCTA**CTACCTTGCAGCTGATGCTGCCGGTCTG**<br>PaqCI recognition sequence PaqCI restriction site<br>**ATAAGATCCAGTTCTACACCCAGTCTGATAGCCTGTCGGGCGTTACTGACGCCCAGT**<br>**TTCACGACCACATTCTTGATGTGAAACTTCACGGTACTCACAGAAATGCCCGTAATA**<br>**GCGGCAATCTCAGCATAGGTTTTGCCCATACTCGCCCAGTACAACACCTCATTTTCA**<br>**CGCGAGGAAAATATCGTTTTGTCCGCGCTCTGATTTAACGCCGGGGCTCGCTCGCCT**<br>**TCGGTACCGGCCAGGCGGTACATCTGCTCGTTAAAATCAATCAGCAGCATCTGCATC**<br>**GTGCCCTGTTCGGCAGCAAGGCGTTGCTCCAGCGCAGTCTGATCGTTGCCTTTAATG**<br>*esaR*<br>**ATCACGGACAACAGAGCAAGGTTGTTCATGTGGTCATGCAGGACATAGGTAAAGCC**<br>**GTTAACGATGTTGTATTGCTTGGATAAAGAGAAAATTTTGGTGAACCGCAGGTCGG**<br>**ACATCAGCGTAATATTCTCATCCCAGGCAAACGGCGAGGTGCGTTTAAAGGCCGTG**<br>**AGAATAACCGGATCGGTCAGCTGAAAGTTGTTAGCGCGGTATAACCTAATCCATTC**<br>**GTCAGGATAACTGGAAATAATCAGAACATTTGAAGGATTTTTTTTGCTCACAACAGT**<br>**GTAAGCGTAATCCGGACTACCCAGCGGAGATAACTTTCTCTGTATGTAAGTCTGAAG**<br>**CGTATCCGTAATGGTCTGGTTTTCCAGGAAGAACGAGAACAT**AGAGACCTCCTTGGAC<br>BsaI restriction site BsaI recognition sequence<br>CTGGTACGTCGCTAGCGGGTAGTGTGACGTGGCGCAGGTGATGGACTTCATGCTGAC<br>GoldenRBS PaqCI restriction site PaqCI recognition sequence |
| $P_{esaR}$ | GATGATGGCGAGCATTCACCTGCGGTACTTAGGTCTCGACATCTAGTACTTTCCTGTGTGACT<br>PaqCI recognition sequence PaqCI restriction site BsaI recognition sequence BsaI restriction site Bba_B0032<br>CTAGTAT**CCGCTAAACAACTGAAGCCATTGTAACCTCTGAATGATTCATTGTAAGTTA**<br>$P_{esaR}$<br>**CTCTTAAGTATCATCTTGCCTGTACTATAGTGCAGGTTAAGTCCACGTTAAGTAAAA**<br>**GAAGCAGC**TCTAGAGATTAAAGAGGAGAAATACTAGATGGTGGCGCAGGTGATGGACTTCA<br>Bba_B0030 PaqCI restriction site PaqCI recognition sequence<br>TGCTGAC |

# MetR:L-homocysteine

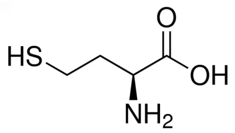


Ligand: L-homocysteine
Source: Sigma- Aldrich 69453
CAS ID: 6027-13-0
Stock: 1 g/L
Solvent: $H_2O$
Storage: -20 °C (2 months)

## Response curve: SynMetR

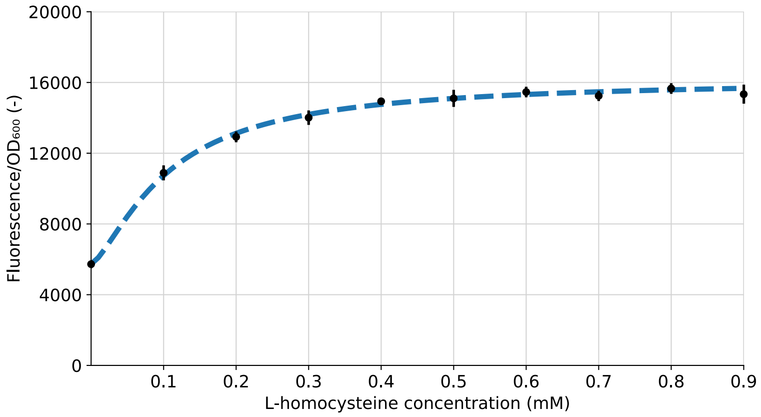


**Hill function**

$$\left(\frac{\text{Fluo}}{OD_{600}}\right)_{cor} = f(C) = a + k\left(\frac{C^n}{C^n + K^n}\right)$$

**Parameters Hill function**

$a_{\text{(a.u)}} = 5.73*10^3 \pm 0.23*10^3$
$k_{\text{(a.u)}} = 1.04*10^4 \pm 0.06*10^4$
$M_{\text{(a.u)}} = 1.62*10^4 \pm 0.06*10^4$
$n = 1.40 \pm 0.31$
$K_{\text{(mM)}} = 1.06*10^{-1} \pm 0.11*10^{-1}$

Error bars depict standard errors over four biological replicates (n=4)
a = the basal normalized fluorescent signal (leaky expression, a.u.)
k = the maximum normalized fluorescent signal relative to a (a.u.)
M = a + k = the maximum normalized fluorescent signal (a.u.)
n = the Hill coefficient (cooperativity, sigmoid character)
K = the Hill constant (TF-ligand affinity, mM)
C = the ligand concentration in the growth medium (mM)

## Metadata

| | |
|---|---|
| Host | *Escherichia coli TOP10 (F- mcrA Δ(mrr-hsdRMS-mcrBC) Φ80lacZΔM15 ΔlacX74 recA1 araD139 Δ(araleu)7697 galU galK rpsL (StrR) endA1 nupG)* |
| Origin | *Salmonella enterica* LT2 |
| Parts | MetR: GenBank AE006468.2 |
| | $P_{metER}$: Intergenic region between *metR* and *metE;* GenBank AE006468.2 |
| Architecture | Synthetic |
| Experiment | 150 µL EZ Rich medium, 96-well black microtiter plate<br>PerkinElmer Ensight, exc. 588nm, emm. 633nm at 30°C<br>Fluorescence after 24h of growth |
| Notes | / |

## MetR:L-homocysteine

| | |
|---|---|
| *metR* | GATGATGGCGAGCATTCACCTGCGGTAGCTA**TTAAATGTTTTTCACATTTTTGAAAAAGTG**<br>PaqCI recognition sequence PaqCI restriction site<br>**CGGTTTCTGGGTATCACGGATAAATTTCAGGTGGGTACAAACATGCAGACGAATGGC**<br>**ATAAATGAAGTTCTTGATGATCAGTTCTTTCTGATCACCATCACGAATGGCTGCATAC**<br>**AGACGTTTCCAAATACCATCACCTAACTTTTTGGTCACCACCAGACCTTGTTTTTCAAA**<br>**GGTATCAACAACCCAATGCGGCAGTGCGGTAATACCCATTTTTGCTGCAACCATCTGA**<br>**ATCAGCAGCAGCGTGTTATTCACGTTTTTAAAGATCGGAATAATACCTGCAGGCTGCA**<br>**GAAAGAATTTCCAGATGTCCAGTTTATCACGTTTCACCGGATAAATCATCAGGATTTC**<br>**GCTGATCAGATCTTCCGGCAGAATATTATGCTTTTTCTGTGCCAGCGGATGATTCGGG**<br>**CTCAGAACCAGACGAACTTCAAAATCAAAGATCGGGAAATAAAACAGGTTGCTACGA**<br>*metR*<br>**GGCAGAACTTCGCTGGTCAGAACAATATCCAGTTTACCCTGCTGCAGGCTCGGCTGC**<br>**GGACTAAAAATCATATCGCTATAAAAGTCGATTTCCACACGAGGCCACTTTTTCTGAA**<br>**AAATCTTCAGAGCCGGTGTCAGCCACTGAATACAGCTATGACATTCAATGGCCAGTTT**<br>**GATAATCATCTGGTGGGTCTTTTTGCAATTCTGGATGGCTTTATGAATTTTCGGCAGG**<br>**ATTTCTTTTGACAGCTGCAGCAGAATTTCACCCTGTGCGGTAAATTTGATCGGGTTGC**<br>**TTTTACGAATAAACAGTTTGAAGCCCAGCTTCTTTTCCAGTTCATTAAACTGATGGCT**<br>**AATTGCGCTCTGGGTCTGATGCAGCTGAATTGCTGCTGCGCTCAGGCTACCGTTATTT**<br>**TTCAGTGCCTGCAGGGTACGCAGATGTTTAATCTCGATCAT**AGAGACCTCCTTGGACCT<br>BsaI restriction site BsaI recognition sequence GoldenRBS<br>GGTACGTCGCTAGCGGGTAGTGTGACGTGGCGCAGGTGATGGACTTCATGCTGAC<br>PaqCI restriction site PaqCI recognition sequence |
| $P_{metER}$ | GATGATGGCGAGCATTCACCTGCGGTACTTACGGTCTCTTCAT**GAGATTCCTTCATATCGAG**<br>PaqCI recognition sequence PaqCI restriction site BsaI recognition sequence BsaI restriction site<br>**AATTAACTATTTGCGCTTGCGAATTTTACTGGATATGAGAATTATAAGATATCTGATTT**<br>$P_{metER}$<br>**AAATAAATCAATATATAAAATTTTATTAATTGATAATTTTATTATGAATGATAAGGAA**<br>**AAAG**ATGGTGGCGCAGGTGATGGACTTCATGCTGAC<br>PaqCI restriction site PaqCI recognition sequence |

# LinR:CHQ

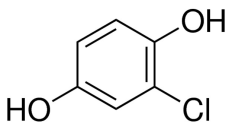


Ligand: Chlorohydroquinone
Source: Sigma- Aldrich 224081
CAS ID: 615-67-8
Stock: 10 g/L
Solvent: $H_2O$
Storage: 2-8 °C (2 months)

## Response curve: SynLinR

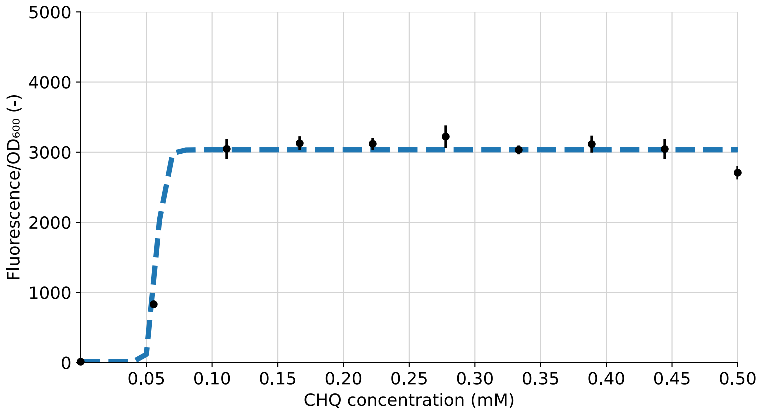


### Hill function

$$\left(\frac{\overline{\text{Fluo}}}{\text{OD}_{600}}\right)_{cor} = f(C) = a + k\left(\frac{C^n}{C^n + K^n}\right)$$

### Parameters Hill function

a (a.u) = $1.22*10^1 \pm 1.13*10^1$
k (a.u) = $3.02*10^3 \pm 0.04*10^3$
M (a.u) = $3.03*10^3 \pm 0.04*10^3$
n = $2.20*10^1 \pm 1.07*10^4$
K (mM) = $5.81*10^{-2} \pm 1.24*10^1$

Error bars depict standard errors over four biological replicates (n=4)
a = the basal normalized fluorescent signal (leaky expression, a.u.)
k = the maximum normalized fluorescent signal relative to a (a.u.)
M = a + k = the maximum normalized fluorescent signal (a.u.)
n = the Hill coefficient (cooperativity, sigmoid character)
K = the Hill constant (TF-ligand affinity, mM)
C = the ligand concentration in the growth medium (mM)

## Metadata

| | |
|---|---|
| Host | *Escherichia coli TOP10 (F- mcrA Δ(mrr-hsdRMS-mcrBC) Φ80lacZΔM15 ΔlacX74 recA1 araD139 Δ(araleu)7697 galU galK rpsL (StrR) endA1 nupG)* |
| Origin | *Sphingobium japonicum* UT26S |
| Parts | LinR: GenBank AP010805.1 |
| | $P_{\text{linER}}$: Intergenic region between *linR* and *linE;* GenBank AP010805.1 |
| Architecture | Synthetic |
| Experiment | 150 µL EZ Rich medium, 96-well black microtiter plate<br>PerkinElmer Ensight, exc. 588nm, emm. 633nm at 30°C<br>Fluorescence after 24h of growth |
| Notes | The Hill function could not be fitted properly to the data. This has resulted in high errors for the n and K parameters. |

# LinR:CHQ

| | |
|---|---|
| *linR* | GATGATGGCGAGCATTCACCTGCGGTAGCTA**TCACATCCGCGCGGACAGCACCTTGTGCA**<br>PaqCI recognition sequence PaqCI restriction site<br>**GGAGCTGTCGCAACCAGCGGTGAGGCGGGTCGTCTTGGTTCCTCGCATGCCAATATT**<br>**GCCTGACCTCAAATGCTTCCATAAGAATTGGCGGCCGCACGGCCCGGAGTCCGTAGA**<br>**CCTCGGCGAGCCGACCAATCACGCGAGCCGGAGCGGTAAGAATAAGGTCCGACTCGA**<br>**AGCAAGCTGCCAACGCCACGAGAAAGCTGCTCGCGACGATCCTGATCCGATCGGGAT**<br>**GGATCTTGTCGAGGAGAGCGCGTTCGACGGCACGATGGGCATGGGCCATACCCTTGG**<br>**TGCTGACCACGATATGATCTGCGGCCATGAAGTCATCTGTCTCACCGGACTTGATGAA**<br>**CGGATGACCTTCCTTTCCGAAGCACAGATATTCCTCCTGGTAGAGCCGTTGCGTCTTG**<br>**ATGCCGGCAACAAGGCTCGGATAGGCGCCGACGGCGATATCGACATGCCCTGTCTCG**<br>*linR*<br>**AGCGCGCTCACCATTTCATCGCCGCTCAGCGTGAGTGCGCGGTAGCTGGTGTGTGGT**<br>**GCCTCGAACCGCGCCGCCGAATGCAACGCCGTCAAAACGACAAGATGGGCGATGTCC**<br>**GATCCGGCAATGATGAATTCGCGCTTAAGACGGTCAGGTGAGAATGTCTGCCCGCCT**<br>**TCAGCCAGGTCGCGACGCAGTTCCAGCAGTTGCTGAACAACCCCGGCGATCGCCTCG**<br>**GCACGTGGTGTCGGCTCCATGCCGTCGCGCGCCCGGACGAGAAGCGGGTCGCCCAAT**<br>**GCTTTGCGCAGGCGCGCCAGCCCATGGCTGGCGGTGGGCTGCGGTAGGTCGAGTTCT**<br>**CGTGCAGCGGCGCTGACGCTGTGCCGCTTTAACAGCGCATCCAAAAGCACCAAGTGC**<br>**CGAAAATCCAGGTCATCTATATTCAT**AGAGACCTCCTTGGACCTGGTACGTCGCTAGCGG<br>BsaI restriction site BsaI recognition sequence GoldenRBS<br>GTAGTGTGACGTGGCGCAGGTGATGGACTTCATGCTGAC<br>PaqCI restriction site PaqCI recognition sequence |
| $P_{linER}$ | GATGATGGCGAGCATTCACCTGCGGTACTTACGGTCTCTTCAT**GCAGCGGCGCTGACGCTGT**<br>PaqCI recognition sequence PaqCI restriction site BsaI recognition sequence BsaI restriction site<br>**GCCGCTTTAACAGCGCATCCAAAAGCACCAAGTGCCGAAAATCCAGGTCATCTATATT**<br>**CACAATCTGAATTGTATCTATGAAGGTCCGCCAATTGTCAAATACTCCCTGATCCCTC**<br>$P_{linER}$<br>**AAACAGGCTGCAGCAGCGATCGTTGTGCAGGTTTGAAGAGACGATCCGCAATCGCAG**<br>**AAAATAGTGGAGAGCGATCTCTAGAGATTAAAGAGGAGAAATACTAG**ATGGTGGCGC<br>PaqCI restriction site<br>AGGTGATGGACTTCATGCTGAC<br>PaqCI recognition sequence |

# LldR:Ca-lactate

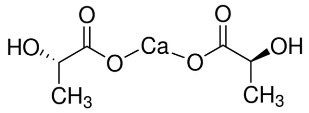


Ligand: Ca-lactate·$5H_2O$
Source: VWR 5551
CAS ID: 5743-47-5
Stock: 25.51 g/L
Solvent: $H_20$
Storage: 2-8 °C (8 months)

## Response curve: SynLldR

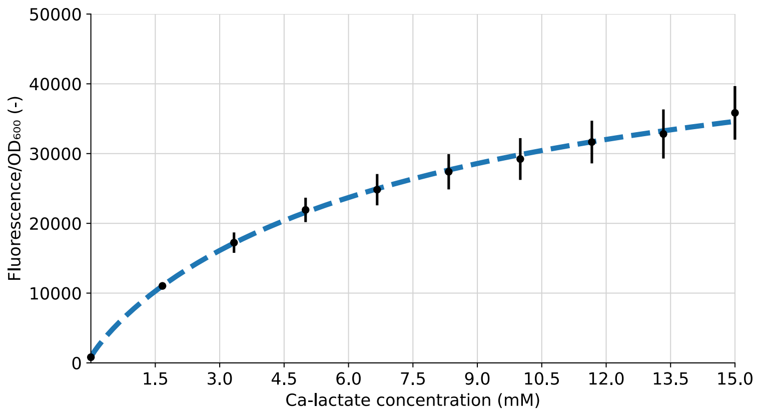


### Hill function

$$\left(\frac{\overline{\text{Fluo}}}{OD_{600}}\right)_{cor} = f\,(C) = a + k\left(\frac{C^n}{C^n + K^n}\right)$$

### Parameters Hill function

$a$ (a.u) = $8.13*10^2 \pm 0.70*10^2$
$k$ (a.u) = $5.41*10^4 \pm 2.38*10^4$
$M$ (a.u) = $5.49*10^4 \pm 2.38*10^4$
$n$ = $8.95*10^{-1} \pm 2.30*10^{-1}$
$K$ (mM) = $8.49 \pm 8.52$

Error bars depict standard errors over four biological replicates (n=4)
a = the basal normalized fluorescent signal (leaky expression, a.u.)
k = the maximum normalized fluorescent signal relative to a (a.u.)
M = a + k = the maximum normalized fluorescent signal (a.u.)
n = the Hill coefficient (cooperativity, sigmoid character)
K = the Hill constant (TF-ligand affinity, mM)
C = the ligand concentration in the growth medium (mM)

## Metadata

| | |
|---|---|
| Host | *Escherichia coli TOP10 (F- mcrA Δ(mrr-hsdRMS-mcrBC) Φ80lacZΔM15 ΔlacX74 recA1 araD139 Δ(araleu)7697 galU galK rpsL (StrR) endA1 nupG)* |
| Origin | *Escherichia coli* |
| Parts | LldR:GenBank EII2917618.1 |
| | $P_{lldPRD}$: BBa_K1847008-RBS0034 |
| Architecture | Synthetic |
| Experiment | 150 µL EZ Rich medium, 96-well black microtiter plate<br>PerkinElmer Ensight, exc. 588nm, emm. 633nm at 30°C<br>Fluorescence after 24h of growth |
| Notes | / |

## LldR:Ca-lactate

| | |
|---|---|
| *lldR* | GATGATGGCGAGCATTCACCTGCGGTAGCTA**TCATGCGTTTTTCTCCCTCGAATGCTCATT**<br>PaqCI recognition sequence PaqCI restriction site<br>**ATGCTCACCGGGCAGGCGGGTAATCCGTGCGTGGCGAGCCTGATCTTCATCGAATCG**<br>**TTTCATGGTGGTGTGAACAAAACTAAGGTGCGCCATCATTGCTTTACGCGCCCCGTCA**<br>**GCATCACCGGCAAAAATGGCGTCAATGACAGCCTGATGTTGTTCGGTCAGTTGTGAA**<br>**AAAACCGGTGGCACCAGATACATCCGCTGACGGCTATGCTTCACTGAGGATTGCAGG**<br>**ACATCGAAGAAACCGCGCATGGTTTGCAGCAGCACGATGTTATGTGAGGCTTCGGCA**<br>**ATCGCCAGATGAAAACGAACGTCCGCTTGTGAGGCGATATCCGGGTCTTCACTTAGC**<br>*lldR*<br>**GTTGCTTCAAAGCAAAGCTGAATCTTTTCTTTGTCGCCAGGTGTGGCGCGCATTGCCG**<br>**CATGCCATGCGGTGCTGGCTTCAATGGCGTAGCGGGCTTCCAGAATATCGAAACTGT**<br>**AATCCGGATCATCGGCCATCAGTGTTTTTAGCGGCTGGACGATGTTTTGCTCCGACCA**<br>**TGTGTCATGACGCCAGCGAATAAACGTCCCGCCGCCGCGTCGACTGAGCAGCACGCC**<br>**TTCACTCACCAGTTTTGCCAGCGCCTCGCGCAGTGAATTACGTGATACGCCGAGTTGC**<br>**ATCGCCAGTTGGCGCTCAGCGGGCAACTTCATGCCCGCTTCCAGGTTTTTTCATCAA**<br>**TCAGCGCCCGCACACGATCGGCAACCTCGTCTGACAGGCGTCTGGGTAAAACAATCA**<br>BsaI restriction site<br>**T**AGAGACCTCCTTGGACCTGGTACGTCGCTAGCGGGTAGTGTGACGTGGCGCAGGTGATGG<br>BsaI recognition sequence GoldenRBS PaqCI restriction site PaqCI recognition sequence<br>ACTTCATGCTGAC |
| $P_{lldPRD}$ | GATGATGGCGAGCATTCACCTGCGGTACTTACGGTCTCTTCAT**AATTGGCCCTACCAATTCT**<br>PaqCI recognition sequence PaqCI restriction site BsaI recognition sequence BsaI restriction site<br>**TCGCTTATCTGACCTCTGGTTCACAATTTCCCAATTAAAACTCACATCAATGTTGACAG**<br>$P_{lldPRD}$<br>**CTAGCTCAGTCCTAGGGATTGTGCTAGCTCATTATCCCTACACAACACAATTGGCAGT**<br>**GCCACTT**TCTAGAGAAAGAGGAGAAATACTAGATGGTGGCGCAGGTGATGGACTTCATGCTG<br>Bba_B0034 PaqCI restriction site PaqCI recognition sequence<br>AC |

# VanR:Vanillic acid

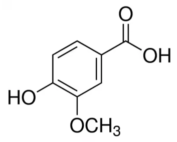


Ligand: Vanillic acid
Source: Sigma- Aldrich 94770
CAS ID: 121-34-6
Stock: 3.75 g/L
Solvent: EtOH
Storage: -20 °C (8 months)

## Response curve: SynVanR

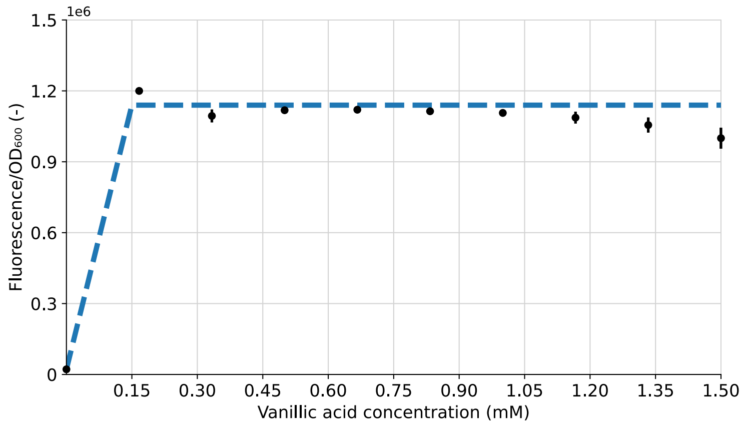


### Hill function

$$\left(\frac{\overline{\text{Fluo}}}{OD_{600}}\right)_{cor} = f(C) = a + k\left(\frac{C^n}{C^n + K^n}\right)$$

### Parameters Hill function

$a_{\text{(a.u)}} = 2.19*10^4$
$k_{\text{(a.u)}} = 2.24*10^6$
$M_{\text{(a.u)}} = 2.26*10^6$
$n = 5.21*10^{-9}$
$K_{\text{(mM)}} = 4.65$

Error bars depict standard errors over four biological replicates (n=4)
a = the basal normalized fluorescent signal (leaky expression, a.u.)
k = the maximum normalized fluorescent signal relative to a (a.u.)
M = a + k = the maximum normalized fluorescent signal (a.u.)
n = the Hill coefficient (cooperativity, sigmoid character)
K = the Hill constant (TF-ligand affinity, mM)
C = the ligand concentration in the growth medium (mM)

## Metadata

| | |
|---|---|
| Host | *Escherichia coli TOP10 (F- mcrA Δ(mrr-hsdRMS-mcrBC) Φ80lacZΔM15 ΔlacX74 recA1 araD139 Δ(araleu)7697 galU galK rpsL (StrR) endA1 nupG)* |
| Origin | *Caulobacter crescentus* |
| Parts | $VanR_{am}$: Evolved, GenBank MH101730.1 |
| | $P_{vanCC}$: Evolved, GenBank MH101730.1 |
| Architecture | Synthetic |
| Experiment | 150 µL EZ Rich medium, 96-well black microtiter plate<br>PerkinElmer Ensight, exc. 588nm, emm. 633nm at 30°C<br>Fluorescence after 20h of growth |
| Notes | The Hill function could not be fitted properly to the data. The Hill function parameter errors could not be calculated. |

## VanR:Vanillic acid

| | |
|---|---|
| *vanR* | GATGATGGCGAGCATTCACCTGCGGTAGCTA**TCAATCTGCACGAATTGACCATGCTGCAC**<br>PaqCI recognition sequence PaqCI restriction site<br>**CCAGCGGTGCGCCTGCGCTTGCTGCTGCTTCAAAAACTTTTGCATTACGAATTGCTGC**<br>**CAGTGCATGATCACGCATAATACGTTCTGCACCTTCGGCATCACCACAGCTAACTGCA**<br>**TCCAGAACTGCCTGATGCTGACGATGTGCTGCCAGCAGATGTTCATATTCGGCAGAC**<br>**AGGTCCATCAGATCCAGGGCCAGTGCACCGGCTGCTGCAAACGGTTCAAAACCATTA**<br>**CGTGCCAGTGCGCTTTCAACTGCACCATTACCTGCTGCGCTAACCAGGGTATCATGAA**<br>**ATGCCTGATTATATGCGGCATAACGATCCAGATCTTCACCATTCAGGCGACCGGCTGC**<br>*vanR*<br>**AAACAGTGCTTCACCTTCTGCAATCAGTACAACAAAACGTGCATGGGTTTCTGCGGTC**<br>**ATACCACGTTCTGCCAGACGACGTGCTGCAAAACCTTCCAGAACACCACGAACTTCAA**<br>**TTGCATCACGAATCTGATCGCTGCTAACACCACGGGCTGCATAACCACGTGCACCCAG**<br>**ACGAACAACCAGACCTTCTTGTTCCAGTGAACGCAGTGCGATACGAACCGGCATACG**<br>**GCTAACACCCAGTGCTGCTGCGGTCGGAATTTCTGCAATACGTTCACCACTTTTGATT**<br>**TCACCGCTTGCAATCATTTTACGCAGTGCCATCATAACACGCTGACCCGGTTTAATAC**<br>**GAGGCATGTCCAT**AGAGACCTCCTTGGACCTGGTACGTCGCTAGCGGGTAGTGTGACGTGG<br>BsaI restriction site BsaI recognition sequence GoldenRBS PaqCI restriction site<br>CGCAGGTGATGGACTTCATGCTGAC<br>PaqCI recognition sequence |
| $P_{vanCC}$ | GATGATGGCGAGCATTCACCTGCGGTACTTACGGTCTCTCCAT**ATTGGATCCAATTGACAGC**<br>PaqCI recognition sequence PaqCI restriction site BsaI recognition sequence BsaI restriction site<br>**TAGCTCAGTCCTAGGTACCATTGGATCCAATAGCTGTCACCGGATGTGCTTTCCGGTC**<br>$P_{vanCC}$<br>**TGATGAGTCCGTGAGGACGAAACAGCCTCTACAAATAATTTTGTTTAA**TACTAGAGAAA<br>GAGGGGAAATACTAGATGGTGGCGCAGGTGATGGACTTCATGCTGAC<br>synthetic RBS PaqCI restriction site PaqCI recognition sequence |

# CueR:$CuSO_4$

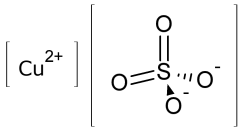


Ligand: $CuSO_4 \cdot 5H_20$
Source: Sigma-Aldrich 209198
CAS ID: 7758-98-7
Stock: 1.2 g/L
Solvent: $H_20$
Storage: -20 °C (8 months)

## Response curve: SynCueR

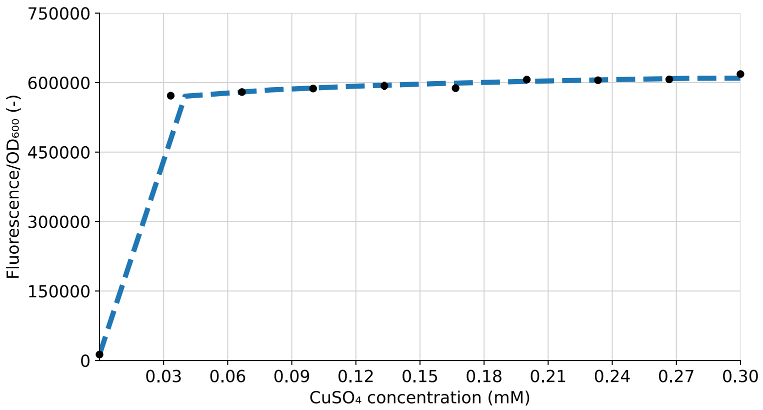


### Hill function

$$\left(\frac{\overline{\text{Fluo}}}{\text{OD}_{600}}\right)_{cor} = f\,(C) = a + k\left(\frac{C^n}{C^n + K^n}\right)$$

### Parameters Hill function

$a$ (a.u) = $1.29*10^4$
$k$ (a.u) = $1.68*10^8$
$M$ (a.u) = $1.68*10^8$
$n$ = $3.48*10^{-2}$
$K$ (mM) = $4.72*10^{69}$

Error bars depict standard errors over four biological replicates (n=4)
a = the basal normalized fluorescent signal (leaky expression, a.u.)
k = the maximum normalized fluorescent signal relative to a (a.u.)
M = a + k = the maximum normalized fluorescent signal (a.u.)
n = the Hill coefficient (cooperativity, sigmoid character)
K = the Hill constant (TF-ligand affinity, mM)
C = the ligand concentration in the growth medium (mM)

## Metadata

| | |
|---|---|
| Host | *Escherichia coli TOP10 (F- mcrA Δ(mrr-hsdRMS-mcrBC) Φ80lacZΔM15 ΔlacX74 recA1 araD139 Δ(araleu)7697 galU galK rpsL (StrR) endA1 nupG)* |
| Origin | *Escherichia coli* K12 |
| Parts | CueR: GenBank EGQ7458477.1 |
| | $P_{copA}$: GenBank CP097884.1 |
| Architecture | Synthetic |
| Experiment | 150 µL EZ Rich medium, 96-well black microtiter plate<br>PerkinElmer Ensight, exc. 588nm, emm. 633nm at 30°C<br>Fluorescence after 24h of growth |
| Notes | The Hill function could not be fitted properly to the data. The Hill function parameter errors could not be calculated. |

# CeuR:CuSO4

| | |
|---|---|
| *ceuR* | GATGATGGCGAGCATTCACCTGCGGTAGCTA**TTACCCTGCGCGATGATGACAACAACCCG**<br>PaqCI recognition sequence PaqCI restriction site<br>**ACAGGTTTTCAATAATCGGACAGTCGGCACTATCGTCGCCCGGACACGCGTTAGCCA**<br>**GCGCCAGCAGTTGGTCCCGCATGCTCTGTAATTCTTCGATATGGCGCTCAATTTCCGC**<br>**AACTTTTTCAAGGGTACGGCGTTTCACATCAGCAGAGTGACGTTGCGGATCGTTGAA**<br>*cueR*<br>**CAAATTGACCAGTTCACCGCTCTCCTCAAGGTTGAACCCTACTTGACGGGCCTGGCGC**<br>**AGTAACGTCAACTCATTGAGGTGCTGTTGCGTATAGGTGCGATAACCATTCTCTGAGC**<br>**GCATCGGCGGAGTAACCAAACCTTTTTCTTCATAAAAGCGGATTGCTTTGCTGGTCAG**<br>**ACCTGTGATCTTCGCCACATCGGAGATGTTTCAT**AGAGACCTCCTTGGACCTGGTACGTCG<br>BsaI restriction site BsaI recognition sequence GoldenRBS<br>CTAGCGGGTAGTGTGACGTGGCGCAGGTGATGGACTTCATGCTGAC<br>PaqCI restriction site PaqCI recognition sequence |
| $P_{copA}$ | GATGATGGCGAGCATTCACCTGCGGTACTTACGGTCTCTTCAT**GGACTTTTACCCGCCTGGT**<br>PaqCI recognition sequence PaqCI restriction site BsaI recognition sequence BsaI restriction site<br>**TTATTAATTTCTTGACCTTCCCCTTGCTGGAAGGTTTAACCTTTATCACAGCCAGTCAA**<br>$P_{copA}$<br>**AACTGTCTTAAAGGAGTGTTTTAGCTGTCACCGGATGTGCTTTCCGGTCTGATGAGTC**<br>**CGTGAGGACGAAACAGCCTCTACAAATAATTTTGTTTAA**TCTAGAGAAAGAGGAGAAAT<br>synthetic RBS<br>ACTAGATGGTGGCGCAGGTGATGGACTTCATGCTGAC<br>PaqCI restriction site PaqCI recognition sequence |

# ZntR:$ZnSO_4$

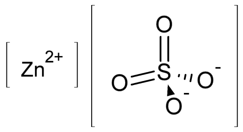


Ligand: $ZnSO_4 \cdot 7H_20$
Source: VWR 1884
CAS ID: 7446-20-0
Stock: 2.2 g/L
Solvent: $H_20$
Storage: -20 °C (8 months)

## Response curve: SynZntR

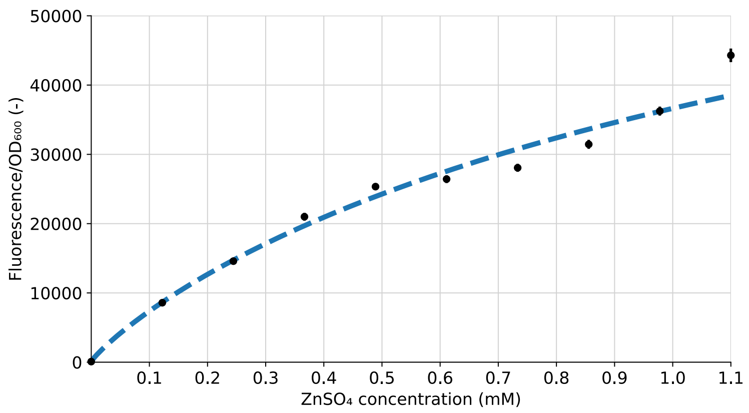


**Hill function**

$$\left(\frac{\overline{\text{Fluo}}}{\text{OD}_{600}}\right)_{cor} = f(C) = a + k\left(\frac{C^n}{C^n + K^n}\right)$$

**Parameters Hill function**

a (a.u) = $8.81 \times 10^{1} \pm 5.24 \times 10^{1}$
k (a.u) = $9.30 \times 10^{4} \pm 3.34 \times 10^{4}$
M (a.u) = $9.31 \times 10^{4} \pm 3.34 \times 10^{4}$
n = $8.82 \times 10^{-1} \pm 0.89 \times 10^{-1}$
K (mM) = $1.64 \pm 1.13$

Error bars depict standard errors over four biological replicates (n=4)
a = the basal normalized fluorescent signal (leaky expression, a.u.)
k = the maximum normalized fluorescent signal relative to a (a.u.)
M = a + k = the maximum normalized fluorescent signal (a.u.)
n = the Hill coefficient (cooperativity, sigmoid character)
K = the Hill constant (TF-ligand affinity, mM)
C = the ligand concentration in the growth medium (mM)

## Metadata

| | |
|---|---|
| Host | *Escherichia coli TOP10 (F- mcrA Δ(mrr-hsdRMS-mcrBC) Φ80lacZΔM15 ΔlacX74 recA1 araD139 Δ(araleu)7697 galU galK rpsL (StrR) endA1 nupG)* |
| Origin | *Escherichia coli* K12 |
| Parts | ZntR: GenBank OSL45746.1 |
| | $P_{zntAR}$: GenBank AP027461.1 |
| Architecture | Synthetic |
| Experiment | 150 µL EZ Rich medium, 96-well black microtiter plate<br>PerkinElmer Ensight, exc. 588nm, emm. 633nm at 30°C<br>Fluorescence after 24h of growth |
| Notes | / |

## ZntR:ZnSO4

| | |
|---|---|
| *zntR* | GATGATGGCGAGCATTCACCTGCGGTAGCTA**TCAACAACCACTCTTAACGCCACTCGCCC**<br>PaqCI recognition sequence PaqCI restriction site<br>**CTTGTTCAAGAGCTTCAAGAATCGAACAATAAACACTGCTATGAGCAGTCCCACAAC**<br>**AGGCATCGTTAAGGCGTTGCAAGGAACGCTGCATACTCTGCAACTCGGCTATCCGTG**<br>**CTTCGACTTCCTGCAATCTTTCCTGCACAATGCCTTTTGACTCCTGACAGGTATGGTGT**<br>**TCAGGATCGATGCGGATCGACAGCAACTCGCGGATCGACTCCAGACTGAAACCTAGT**<br>*zntR*<br>**TGTCTGGCATGGCGGATAAATTTCAATCGCTGGAGATCGCTTTCGGTATATAGGCGA**<br>**AACCCACCTTCAGTACGCACTTCATGCTCCATCATCTGCTGTTTTTCGTAATAACGAAT**<br>**CGTGTCGGGTGTTACTTCCGCCATTTTTGCCAGCTCACCAATGCGATACAT**AGAGACCT<br>BsaI restriction site BsaI recognition sequence<br>CCTTGGACCTGGTACGTCGCTAGCGGGTAGTGTGACGTGGCGCAGGTGATGGACTTCATGCT<br>GoldenRBS PaqCI restriction site PaqCI recognition sequence<br>GAC |
| $P_{zntAR}$ | GATGATGGCGAGCATTCACCTGCGGTACTTACGGTCTCTACAT**TAAAACTTGACTCTGGAGT**<br>PaqCI recognition sequence PaqCI restriction site BsaI recognition sequence BsaI restriction site<br>**CGACTCCAGAGTGTATCCTTCGGTTAATGAGAAAAAACTTAACCGGAGGATGCC**ATGG<br>$P_{zntAR}$ PaqCI restriction site<br>TGGCGCAGGTGATGGACTTCATGCTGAC<br>PaqCI recognition sequence |

# LinR:CHQ

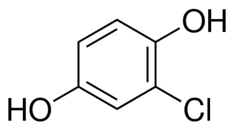

Ligand: Chlorohydroquinone
Source: Sigma- Aldrich 224081
CAS ID: 615-67-8
Stock: 10 g/L
Solvent: $H_2O$
Storage: 2-8 °C (2 months)

## Response curve: NatLinR

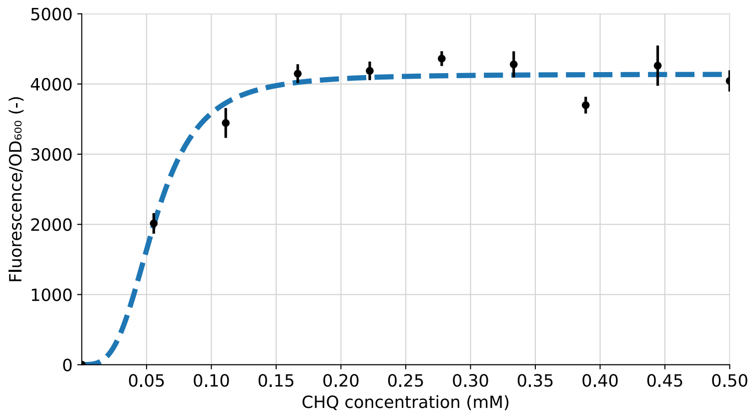


### Hill function

$$\left(\frac{\overline{\text{Fluo}}}{\text{OD}_{600}}\right)_{cor} = f(C) = a + k\left(\frac{C^n}{C^n + K^n}\right)$$

### Parameters Hill function

$a_{(a.u)} = 1.40 \pm 1.84$

$k_{(a.u)} = 4.14*10^3 \pm 0.07*10^3$

$M_{(a.u)} = 4.14*10^3 \pm 0.07*10^3$

$n = 3.29 \pm 0.80$

$K_{(mM)} = 5.68*10^{-2} \pm 0.25*10^{-2}$

Error bars depict standard errors over four biological replicates (n=4)
a = the basal normalized fluorescent signal (leaky expression, a.u.)
k = the maximum normalized fluorescent signal relative to a (a.u.)
M = a + k = the maximum normalized fluorescent signal (a.u.)
n = the Hill coefficient (cooperativity, sigmoid character)
K = the Hill constant (TF-ligand affinity, mM)
C = the ligand concentration in the growth medium (mM)

## Metadata

| | |
|---|---|
| Host | *Escherichia coli TOP10 (F- mcrA Δ(mrr-hsdRMS-mcrBC) Φ80lacZΔM15 ΔlacX74 recA1 araD139 Δ(araleu)7697 galU galK rpsL (StrR) endA1 nupG)* |
| Origin | *Sphingobium japonicum* UT26S |
| Parts | LinR: GenBank AP010805.1 |
| | $P_{linER}$: Intergenic region between *linR* and *linE;* GenBank AP010805.1 |
| Architecture | Natural |
| Experiment | 150 µL EZ Rich medium, 96-well black microtiter plate<br>PerkinElmer Ensight, exc. 588nm, emm. 633nm at 30°C<br>Fluorescence after 24h of growth |
| Notes | / |

# LinR:CHQ

| | |
|---|---|
| *linR* | GATGATGGCGAGCATTCACCTGCGGTAGCTA**TCACATCCGCGCGGACAGCACCTTGTGCA**<br>PaqCI recognition sequence PaqCI restriction site<br>**GGAGCTGTCGCAACCAGCGGTGAGGCGGGTCGTCTTGGTTCCTCGCATGCCAATATT**<br>**GCCTGACCTCAAATGCTTCCATAAGAATTGGCGGCCGCACGGCCCGGAGTCCGTAGA**<br>**CCTCGGCGAGCCGACCAATCACGCGAGCCGGAGCGGTAAGAATAAGGTCCGACTCGA**<br>**AGCAAGCTGCCAACGCCACGAGAAAGCTGCTCGCGACGATCCTGATCCGATCGGGAT**<br>**GGATCTTGTCGAGGAGAGCGCGTTCGACGGCACGATGGGCATGGGCCATACCCTTGG**<br>**TGCTGACCACGATATGATCTGCGGCCATGAAGTCATCTGTCTCACCGGACTTGATGAA**<br>**CGGATGACCTTCCTTTCCGAAGCACAGATATTCCTCCTGGTAGAGCCGTTGCGTCTTG**<br>**ATGCCGGCAACAAGGCTCGGATAGGCGCCGACGGCGATATCGACATGCCCTGTCTCG**<br>*linR*<br>**AGCGCGCTCACCATTTCATCGCCGCTCAGCGTGAGTGCGCGGTAGCTGGTGTGTGGT**<br>**GCCTCGAACCGCGCCGCCGAATGCAACGCCGTCAAAACGACAAGATGGGCGATGTCC**<br>**GATCCGGCAATGATGAATTCGCGCTTAAGACGGTCAGGTGAGAATGTCTGCCCGCCT**<br>**TCAGCCAGGTCGCGACGCAGTTCCAGCAGTTGCTGAACAACCCCGGCGATCGCCTCG**<br>**GCACGTGGTGTCGGCTCCATGCCGTCGCGCGCCCGGACGAGAAGCGGGTCGCCCAAT**<br>**GCTTTGCGCAGGCGCGCCAGCCCATGGCTGGCGGTGGGCTGCGGTAGGTCGAGTTCT**<br>**CGTGCAGCGGCGCTGACGCTGTGCCGCTTTAACAGCGCATCCAAAAGCACCAAGTGC**<br>**CGAAAATCCAGGTCATCTATATTCAT**AGAGACCTCCTTGGACCTGGTACGTCGCTAGCGG<br>BsaI restriction site BsaI recognition sequence GoldenRBS<br>GTAGTGTGACGTGGCGCAGGTGATGGACTTCATGCTGAC<br>PaqCI restriction site PaqCI recognition sequence |
| $P_{linER}$ | GATGATGGCGAGCATTCACCTGCGGTACTTACGGTCTCTTCAT**GCAGCGGCGCTGACGCTGT**<br>PaqCI recognition sequence PaqCI restriction site BsaI recognition sequence BsaI restriction site<br>**GCCGCTTTAACAGCGCATCCAAAAGCACCAAGTGCCGAAAATCCAGGTCATCTATATT**<br>**CACAATCTGAATTGTATCTATGAAGGTCCGCCAATTGTCAAATACTCCCTGATCCCTC**<br>$P_{linER}$<br>**AAACAGGCTGCAGCAGCGATCGTTGTGCAGGTTTGAAGAGACGATCCGCAATCGCAG**<br>**AAAATAGTGGAGAGCGATCTCTAGAGATTAAAGAGGAGAAATACTAG**ATGGTGGCGC<br>PaqCI restriction site<br>AGGTGATGGACTTCATGCTGAC<br>PaqCI recognition sequence |

# MetR:L-homocysteine

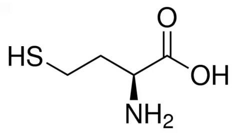

Ligand: L-homocysteine
Source: Sigma- Aldrich 69453
CAS ID: 6027-13-0
Stock: 1 g/L
Solvent: $H_2O$
Storage: -20 °C (2 months)

## Response curve: NatMetR

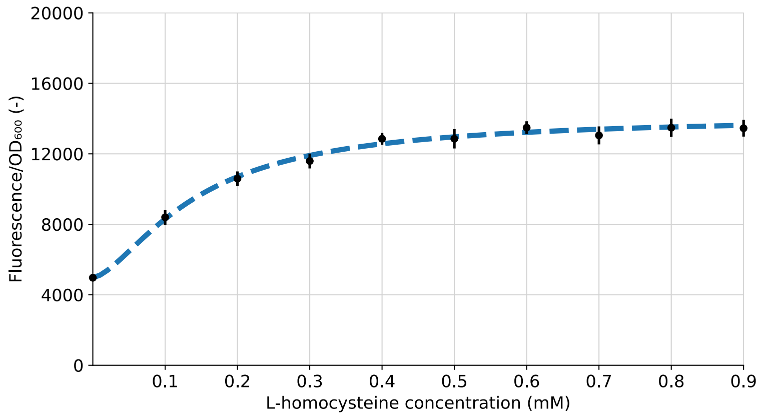


### Hill function

$$\left(\frac{\overline{\text{Fluo}}}{OD_{600}}\right)_{cor} = f(C) = a + k\left(\frac{C^n}{C^n + K^n}\right)$$

### Parameters Hill function

a (a.u) = $4.97*10^3 \pm 0.13*10^3$
k (a.u) = $9.15*10^3 \pm 0.64*10^3$
M (a.u) = $1.41*10^4 \pm 0.07*10^4$
n = $1.55 \pm 0.35$
K (mM) = $1.43*10^{-1} \pm 0.18*10^{-1}$

Error bars depict standard errors over four biological replicates (n=4)
a = the basal normalized fluorescent signal (leaky expression, a.u.)
k = the maximum normalized fluorescent signal relative to a (a.u.)
M = a + k = the maximum normalized fluorescent signal (a.u.)
n = the Hill coefficient (cooperativity, sigmoid character)
K = the Hill constant (TF-ligand affinity, mM)
C = the ligand concentration in the growth medium (mM)

## Metadata

| | |
|---|---|
| Host | *Escherichia coli TOP10 (F- mcrA Δ(mrr-hsdRMS-mcrBC) Φ80lacZΔM15 ΔlacX74 recA1 araD139 Δ(araleu)7697 galU galK rpsL (StrR) endA1 nupG)* |
| Origin | *Salmonella enterica* LT2 |
| Parts | MetR: GenBank AE006468.2 |
| | $P_{metER}$: Intergenic region between *metR* and *metE;* GenBank AE006468.2 |
| Architecture | Natural |
| Experiment | 150 µL EZ Rich medium, 96-well black microtiter plate<br>PerkinElmer Ensight, exc. 588nm, emm. 633nm at 30°C<br>Fluorescence after 24h of growth |
| Notes | / |

# MetR:L-homocysteine

| | |
|---|---|
| *metR* | GATGATGGCGAGCATTCACCTGCGGTAGCTA**TTAAATGTTTTTCACATTTTTGAAAAAGTG**<br>PaqCI recognition sequence PaqCI restriction site<br>**CGGTTTCTGGGTATCACGGATAAATTTCAGGTGGGTACAAACATGCAGACGAATGGC**<br>**ATAAATGAAGTTCTTGATGATCAGTTCTTTCTGATCACCATCACGAATGGCTGCATAC**<br>**AGACGTTTCCAAATACCATCACCTAACTTTTTGGTCACCACCAGACCTTGTTTTTCAAA**<br>**GGTATCAACAACCCAATGCGGCAGTGCGGTAATACCCATTTTTGCTGCAACCATCTGA**<br>**ATCAGCAGCAGCGTGTTATTCACGTTTTTAAAGATCGGAATAATACCTGCAGGCTGCA**<br>**GAAAGAATTTCCAGATGTCCAGTTTATCACGTTTCACCGGATAAATCATCAGGATTTC**<br>**GCTGATCAGATCTTCCGGCAGAATATTATGCTTTTTCTGTGCCAGCGGATGATTCGGG**<br>**CTCAGAACCAGACGAACTTCAAAATCAAAGATCGGGAAATAAAACAGGTTGCTACGA**<br>*metR*<br>**GGCAGAACTTCGCTGGTCAGAACAATATCCAGTTTACCCTGCTGCAGGCTCGGCTGC**<br>**GGACTAAAAATCATATCGCTATAAAAGTCGATTTCCACACGAGGCCACTTTTTCTGAA**<br>**AAATCTTCAGAGCCGGTGTCAGCCACTGAATACAGCTATGACATTCAATGGCCAGTTT**<br>**GATAATCATCTGGTGGGTCTTTTTGCAATTCTGGATGGCTTTATGAATTTTCGGCAGG**<br>**ATTTCTTTTGACAGCTGCAGCAGAATTTCACCCTGTGCGGTAAATTTGATCGGGTTGC**<br>**TTTTACGAATAAACAGTTTGAAGCCCAGCTTCTTTTCCAGTTCATTAAACTGATGGCT**<br>**AATTGCGCTCTGGGTCTGATGCAGCTGAATTGCTGCTGCGCTCAGGCTACCGTTATTT**<br>**TTCAGTGCCTGCAGGGTACGCAGATGTTTAATCTCGATCAT**AGAGACCTCCTTGGACCT<br>BsaI restriction site BsaI recognition sequence GoldenRBS<br>GGTACGTCGCTAGCGGGTAGTGTGACGTGGCGCAGGTGATGGACTTCATGCTGAC<br>PaqCI restriction site PaqCI recognition sequence |
| $P_{metER}$ | GATGATGGCGAGCATTCACCTGCGGTACTTACGGTCTCTTCAT**GAGATTCCTTCATATCGAG**<br>PaqCI recognition sequence PaqCI restriction site BsaI recognition sequence BsaI restriction site<br>**AATTAACTATTTGCGCTTGCGAATTTTACTGGATATGAGAATTATAAGATATCTGATTT**<br>$P_{metER}$<br>**AAATAAATCAATATATAAAATTTTATTAATTGATAATTTTATTATGAATGATAAGGAA**<br>**AAAG**ATGGTGGCGCAGGTGATGGACTTCATGCTGAC<br>PaqCI restriction site PaqCI recognition sequence |
